# Electrocatalytic upcycling of polyethylene terephthalate to commodity chemicals and H_2_ fuel

**DOI:** 10.1038/s41467-021-25048-x

**Published:** 2021-08-17

**Authors:** Hua Zhou, Yue Ren, Zhenhua Li, Ming Xu, Ye Wang, Ruixiang Ge, Xianggui Kong, Lirong Zheng, Haohong Duan

**Affiliations:** 1 Department of Chemistry, Tsinghua University, Beijing, China; 2 State Key Laboratory of Chemical Resource Engineering, College of Chemistry, Beijing University of Chemical Technology, Beijing, China; 3 Institute of High Energy Physics, The Chinese Academy of Sciences, Beijing, China

**Keywords:** Sustainability, Electrocatalysis, Renewable energy, Nanoparticles

## Abstract

Plastic wastes represent a largely untapped resource for manufacturing chemicals and fuels, particularly considering their environmental and biological threats. Here we report electrocatalytic upcycling of polyethylene terephthalate (PET) plastic to valuable commodity chemicals (potassium diformate and terephthalic acid) and H_2_ fuel. Preliminary techno-economic analysis suggests the profitability of this process when the ethylene glycol (EG) component of PET is selectively electrooxidized to formate (>80% selectivity) at high current density (>100 mA cm^−2^). A nickel-modified cobalt phosphide (CoNi_0.25_P) electrocatalyst is developed to achieve a current density of 500 mA cm^−2^ at 1.8 V in a membrane-electrode assembly reactor with >80% of Faradaic efficiency and selectivity to formate. Detailed characterizations reveal the in-situ evolution of CoNi_0.25_P catalyst into a low-crystalline metal oxy(hydroxide) as an active state during EG oxidation, which might be responsible for its advantageous performances. This work demonstrates a sustainable way to implement waste PET upcycling to value-added products.

## Abstract (web summary)

Plastic upcycling to value-added products is of great interests. Here the authors investigate a nickel-cobalt phosphide electrocatalyst for electroreforming of polyethylene terephthalate plastic toward valuable potassium diformate, terephthalic acid, and H_2_ fuel.

## Introduction

Over 8 billion tons of plastics have been produced to date, and 79% of them are discarded and accumulated in landfills or aquatic systems^1,2^, representing a severe environmental and biological threat^3–6^. Plastic reclaim is essential for non-renewable resource saving, thereby reducing CO_2_ emission, according to the circular economy principle^7,8^. Conventional plastic recycling strategies (e.g., mechanical methods) had limited success (<10% recycling rate) and the reproduced materials are suffering from inferior properties compared with the virgin plastic, and such process is often called downcycling model^1,5^. In this respect, chemical reclaim provides an alternative route to get more value from wastes by catalytically processing them to high-quality monomer subunits^5,9–11^ or upcycling into value-added products^7,12^. The success of these approaches would rely on the efficiency and selectivity of the catalysts and also the sustainability and profitability of the process.

**Fig. 1:**
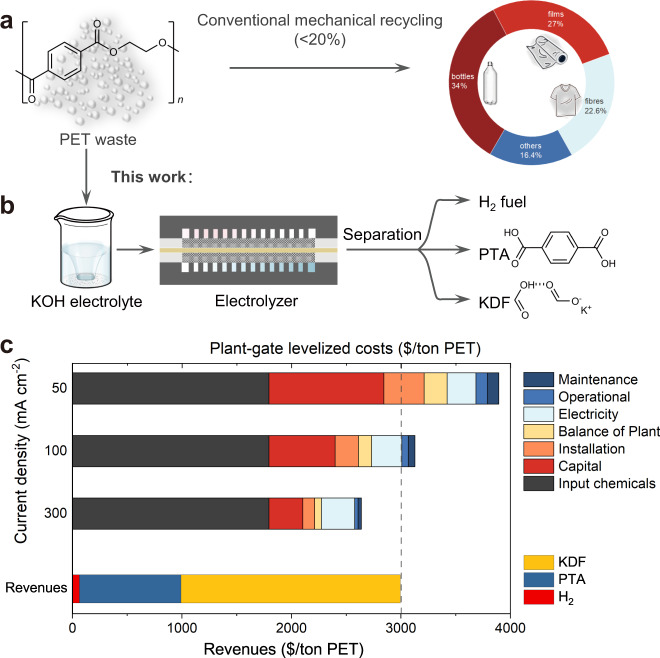
Conceptual design. **a** Conventional route for PET recycling. **b** Electrocatalytic PET upcycling to commodity chemicals and H_2_ fuel (Route I). **c** Techno-economic analysis (TEA) of Route I at different current density.

Polyethylene terephthalate (PET) is produced ~70 million tons annually for packaging and textiles^10^, but only a small fraction (<20%) of them are recycled mainly via mechanical method (Fig. 1a)^5^. Thermal recycling approaches (such as, hydrogenolysis^13^ and glycolysis^14^) lead to the recovery of monomers (terephthalic acid (PTA) or bis(2‐hydroxyethyl) terephthalate) under elevated temperatures. The polyester nature of PET makes it readily decomposed into its monomers under mild conditions catalyzed by base^2,11^ or hydrolases^10^, which can be further transformed into valuable products. Recently, Erwin and co-workers reported a photoreforming strategy for converting PET waste into clean H_2_ fuel and oxygenates (i.e., formate, glyoxal, and acetate) under mild conditions^2,15^. The ethylene glycol (EG) component of PET is readily oxidized by photogenerated holes which improves H_2_ production rate from water. Despite the well-established methodology, the process still suffers from low spatial productivity and poor selectivity towards a single high-value oxidation product (Supplementary Table 1)^16,17^.

Electrocatalysis can be powered by renewable energy (solar, wind, and hydro) that represents a sustainable and attractive strategy to generate clean H_2_ from water at cathode and value-added oxygenates from organic compounds at anode under mild conditions^18–21^. It has obtained great advancements in efficient and selective transformation of various organic compounds, such as simple alcohols and renewable biomass-derived oxygenates, and varieties of valuable carbonyl chemicals such as formate^22,23^, acetate^24,25^, 2,5-furandicarboxylate^26^, adipate^19^ can be obtained. However, the electrocatalytic reforming of PET waste into valuable products is largely unexplored.

Here, we show an electrocatalytic strategy for PET waste upcycling to commodity chemicals of potassium diformate (KDF) and PTA paired with H_2_ production using a bifunctional CoNi_0.25_P electrocatalyst in KOH electrolyte (Fig. 1b). The PET is digested in alkaline solution to give its monomers including PTA and EG, and the latter is selectively (>90%) undergoing C–C cleavage to formate over anodic CoNi_0.25_P catalyst in electrolyzer, alongside the H_2_ generation over the same catalyst at cathode. Subsequently, formic acid is used as an acidifier of the PET electrolyte for PTA precipitation and regeneration by filtration, and the resulting liquid stream is transformed into solid KDF by concentration and crystallization. Preliminary techno-economic analysis (TEA) estimates the net revenues of ~$350 for upcycling per tonne of waste PET under commercially relevant current density (>300 mA cm^−2^). Comprehensive analysis of the material after reactions reveal the formation of metal phosphide/oxy(hydroxide) core–shell structure during cathodic hydrogen evolution reaction (HER) and a complete reconstruction of phosphide to low-crystalline oxy(hydroxide) analogue under anodic conditioning, which might be responsible for its high catalytic performance.

## Results

### Conceptual design and techno-economic analysis

One key factor for the success of electrocatalytic PET reclaim is efficiently and selectively deriving value-added products to compensate the costs of the process^20,27^. Formic acid and formate are important industrial chemicals mainly used in animal feed (34%) with increasing demand (Supplementary Fig. 1). In contrast, their derived functional commodity—KDF, known as Formi™—is an emerging and safe growth promoter for animals with the ban of antibiotic additives for feed^28,29^. In this context, we conceived an integrated process for PET waste upcycling toward targeted commodity chemicals (i.e., KDF, PTA) and clean fuel (H_2_), denoted as Route I. As illustrated in Fig. 1b, the process is mainly composed of three steps: (i) KOH-catalyzed PET hydrolysis (Eq. 1), (ii) electroreforming (ER) of PET hydrolysate (Eq. 2), including EG oxidation (Eq. 3) and paired HER (Eq. 4), and (iii) formic acid-assisted products (KDF, PTA) separation (Supplementary Fig. 2).

1 $${{{{{\rm{Hydrolysis}}}}}}\!:\;({{{{{\rm{PET}}}}}})_{n}+(2n-1){{{{{{\rm{H}}}}}}}_{2}{{{{{\rm{O}}}}}}\!\to\!n{{{{{{\rm{C}}}}}}}_{8}{{{{{{\rm{H}}}}}}}_{6}{{{{{{\rm{O}}}}}}}_{4}({{{{{\rm{PTA}}}}}})+n{{{{{{\rm{C}}}}}}}_{2}{{{{{{\rm{H}}}}}}}_{6}{{{{{{\rm{O}}}}}}}_{2}({{{{{\rm{EG}}}}}})$$

2 $${{{{{\rm{Electroreforming}}}}}}\!:\; {{{{{\rm{C}}}}}}_{2}{{{{{\rm{H}}}}}}_{6}{{{{{{\rm{O}}}}}}}_{2}+{2{{{{{\rm{OH}}}}}}}^{-}\to {2{{{{{\rm{HCOO}}}}}}}^{-}+3{{{{{{\rm{H}}}}}}}_{2}$$

3 $${{{{{\rm{Anode}}}}}}\!:\; {{{{{\rm{C}}}}}}_{2}{{{{{\rm{H}}}}}}_{6}{{{{{{\rm{O}}}}}}}_{2}+{8{{{{{\rm{OH}}}}}}}^{-}\to {2{{{{{\rm{HCOO}}}}}}}^{-}+6{{{{{{\rm{H}}}}}}}_{2}{{{{{\rm{O}}}}}}+6{{{{{{\rm{e}}}}}}}^{-}\,$$

4 $${{{{{\rm{Cathode}}}}}}\!:\;6{{{{{{\rm{H}}}}}}}_{2}{{{{{\rm{O}}}}}}+6{{{{{{\rm{e}}}}}}}^{-}\to {3{{{{{\rm{H}}}}}}}_{2}+6{{{{{{\rm{OH}}}}}}}^{-}$$

We then conducted a preliminary TEA to investigate the feasibility of this conceptual system using a model (Supplementary Figs. 2 and 3) adapted from literatures, reported by Sargent and co-workers^30–32^. It suggests that the profitability of the process depends on the cost of renewable electricity, Faradaic efficiency to formate (FE_formate_), and operating current density (Supplementary Fig. 4). With the development of renewable electricity, its cost continues to plummet to below^31^ 10 cents  kWh^−1^. In this context, ER of PET is of economic possibility when the electrocatalyst achieves high FE_formate_ (>80%) and high current density (>100 mA cm^−2^) (Fig. 1c and Supplementary Fig. 4).

Conventionally, the alkali PET hydrolysate is neutralized by mineral acids (e.g., H_2_SO_4_) for PTA precipitation, denoted as Route II (Supplementary Fig. 5a), if one only targets the recovery of PTA and EG monomers. The subsequent separation of EG and K_2_SO_4_ step requires more distillation equipment and intense energy because of the high boiling point (197 °C) and excellent water solubility of EG. In contrast, formic acid is used as the acidifier for PTA separation and KDF production in Route I, simplifying the separation process and improving the revenue of products. Even though the costs related to electrolysis is eliminated in Route II, TEA suggests that it is without profitability owing to the relatively low returns of the products (PTA, EG, and K_2_SO_4_) (Supplementary Fig. 5b). Similarly, there is low economic possibility for Route III (Supplementary Fig. 6) toward PTA, formic acid, H_2_, and K_2_SO_4_ as final products when using H_2_SO_4_ for separating products from the PET electrolyte.

Overall, the preliminary TEA result indicates the economic potential of the electrocatalytic upcycling of PET waste to KDF, PTA, and H_2_, and highlights the requirement of advanced and cost-effective electrocatalysts to realize EG oxidation to formate, ideally a bifunctional electrocatalyst for both the reactions, at commercially relevant current density (>100 mA cm^−2^) in high FE_formate_ and selectivity (>80%).

### Optimization of electrocatalyst for HER and EG oxidation

Transition metal-based phosphides (especially for earth-abundant Co and Ni) are demonstrated to be efficient electrocatalyst in various reactions, including HER, oxygen evolution reaction (OER), and organic transformations^33–35^. To develop a bifunctional electrocatalyst for HER and EG oxidation, we synthesized a series of Co and Ni phosphides supported on nickel foam (NF) including CoNi_*x*_P/NF (*x* = 0, 0.1, 0.25, 0.5, the feed atomic ratios of Ni/Co) and Ni_2_P/NF via tandem electrodeposition and phosphidation as illustrated in Supplementary Fig. 7. The nanoarray is composed of α-phase metal hydroxide nanosheets that was obtained by electrodeposition (Supplementary Fig. 8) with following phosphidation to give metal phosphides (Supplementary Fig. 9 and Supplementary Table 2). For comparison, metal hydroxide-derived oxy(hydroxide) and oxide were also prepared (Supplementary Figs. 7, 10, and 11).

**Fig. 2:**
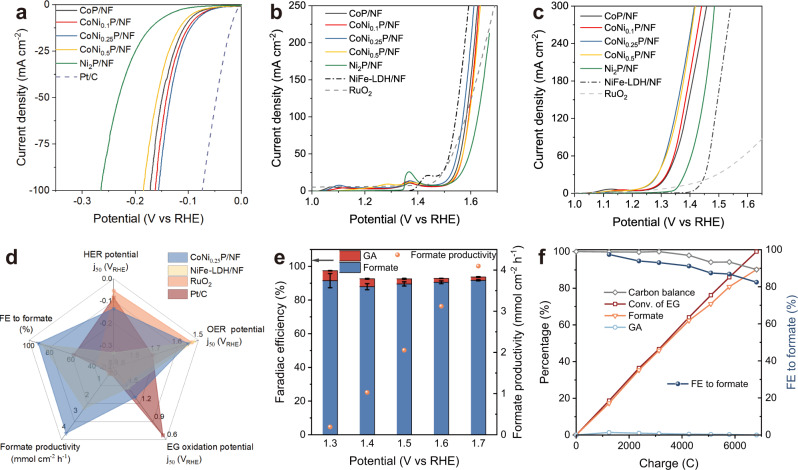
Electrochemical evaluation of CoNi_*x*_P and Ni_2_P catalysts. **a** HER polarization curves (100% iR corrected). **b**, **c** LSV curves (85% iR corrected) for OER (**b**) and EG oxidation (**c**). **d** Comparison of the catalytic performances of CoNi_0.25_P/NF and known catalysts. **e** FE and productivity of formate on CoNi_0.25_P/NF at different potentials. Error bars correspond to the standard deviation of three measurements. **f** Kinetic curves for EG transformation at 1.5 V vs RHE. Reaction conditions: all experiments were performed in 1 M KOH in H-cell separated by AEM, the scan rate is 5 mV s^−1^ for all polarization curves, the EG concentration is 0.3 M.

The as-prepared samples were evaluated for HER in an alkaline electrolyte (1 M KOH). As shown in Fig. 2a, the CoNi_0.25_P/NF exhibits the best performance among the samples with a low Tafel slope of 58.1 mV decade^−1^ (Supplementary Fig. 12), representing an advantageous material compared to the reported non-noble metal phosphides for HER (Supplementary Table 2). The improved HER activity of CoNi_0.25_P/NF can be attributed to the decreased charge transfer resistance and increased electrochemically active surface area (Supplementary Fig. 13). Subsequently, we tested the oxidative performance of CoNi_*x*_P/NF and Ni_2_P/NF electrocatalysts in 1 M KOH without or with 0.3 M EG. In the absence of EG, all the electrocatalysts exhibit comparative OER activity with similar onset potential around 1.51 V vs RHE (Fig. 2b). Whereas the onset potential of all the electrocatalysts shifts toward lower potential (1.22–1.33 V vs RHE) after introducing 0.3 M EG into electrolyte (Fig. 2c), indicating EG oxidation is thermodynamically more favorable than OER. Among these materials, CoNi_0.25_P/NF and CoNi_0.5_P/NF outperform other self-prepared Co, Ni phosphides and report efficient electrocatalysts (NiFe-LDH and commercial RuO_2_) with lower onset potential and higher current density for EG oxidation under the same conditions (Fig. 2c). Together with their HER performances, the CoNi_0.25_P/NF was selected as both the cathodic and anodic catalyst for further evaluation.

Scanning electron microscopy (SEM) and elemental mapping show the nanoarray structure of CoNi_0.25_P with homogenous distribution of Co, Ni, and P on matrix (Supplementary Fig. 14a–c). Furthermore, high-resolution transmission electron microscopy (HRTEM) reveals that small Ni_2_P nanoparticles are inter-connected with CoP particles, forming CoP–Ni_2_P heterojunctions in CoNi_0.25_P material. In addition, X-ray diffraction (XRD) Rietveld refinement and extended X-ray absorption fine-structure spectroscopy (EXAFS) fitting were performed. They reveal the co-existence of CoP and Ni_2_P phase in CoNi_0.25_P (Supplementary Fig. 15), with an atomic Co:Ni ratio of approximately 1:0.13 (Supplementary Table 3), which is consistent with the results of inductively coupled plasma-atomic emission spectrometry (ICP-AES) and energy dispersive X-ray (EDX) analysis (Supplementary Table 4).

We then systematically compared the catalytic performances of CoNi_0.25_P/NF with known electrocatalysts (i.e., Pt/C, RuO_2_, NiFe-LDH/NF) in EG oxidation and HER (Supplementary Figs. 16 and 17), and the results are summarized in Fig. 2d. Specifically, CoNi_0.25_P/NF displays the highest FE_formate_ (91.3%) for selective EG oxidation with an excellent spatial productivity of 4.01 mmol cm^−2^ h^−1^ at 1.7 V vs RHE, as well as good HER activity. For precious metal catalysts (i.e., Pt/C and RuO_2_), they fail to achieve efficient and selective EG transformation into formate, although they display excellent HER performances. Despite the ultra-low potential (0.67 V vs RHE) required for Pt/C to achieve a current density of 50 mA cm^−2^ in EG oxidation (Fig. 2d), the main detected product is glycollate (GA, FE = 68.8% at 1.0 V vs RHE) (Supplementary Figs. 16d and 17), revealing the low reactivity of Pt/C for oxidative C–C bond cleavage. It is well in agreement with the catalytic performances of noble metals (Pt, Au, Pd) for the oxidation of alcohols to aldehydes, in which the aldehydes are readily undergo an intermolecular Cannizzaro rearrangement to produce corresponding carboxylates and alcohols in base media^36,37^. NiFe-LDH/NF displays high activity for alkaline OER (Fig. 2b), consistent with literature^38^, whereas it is inferior to CoNi_0.25_P/NF for breaking C–C bond in EG to formate (Fig. 2d and Supplementary Fig. 17). This result suggests that the active catalyst for OER may not be efficient for oxidative C–C cleavage in EG. In addition, the lower activity of CoNi_0.25_(OH)_2_/NF-derived oxy(hydroxide) and oxide compared with its phosphide (CoNi_0.25_P/NF) indicates the important role of phosphorization in improving the activity and selectivity for cutting C–C in EG to formate (Supplementary Figs.17 and 18a). This may be attributed to that the phosphorization can accelerate the reconstruction of materials toward active catalyst^39^, and lower the charge transfer resistance (Supplementary Fig. 18b).

### Electrocatalytic EG oxidation over CoNi_0.25_P/NF

The EG transformation over CoNi_0.25_P/NF anode was evaluated in a three-electrode H-cell system separated by an anion exchange membrane (AEM). As shown in Supplementary Fig. 19a, large numbers of H_2_ bubbles are released from the cathode surface, while no bubble is observed on the anode in 1 M KOH with EG under 1.7 V vs RHE. By contrast, O_2_ bubbles are generated over the anode in a blank 1 M KOH anolyte under the same potential (Supplementary Fig. 19b). This is due to the fact that EG oxidation is thermodynamically more favorable than OER. The products of EG conversion were analyzed by high-performance liquid chromatography (HPLC). As shown in Supplementary Fig. 20a, formate is the dominant product with ~90% of FE under a broad potential window (1.3–1.7 V vs RHE, Fig. 2e), revealing the high selectivity of EG transformation over CoNi_0.25_P/NF. Notably, the CoNi_0.25_P/NF achieves 91.7% of FE_formate_ and 4.1 mmol cm^−2^ h^−1^ of formate productivity at a commercially relevant current density (~350 mA cm^−2^) under 1.7 V vs RHE.

Additionally, HPLC analysis was adopted to track the dynamic conversion of EG over CoNi_0.25_P/NF in a batch reaction. A small fraction of GA is the only observed product during EG oxidation (Fig. 2f and Supplementary Fig. 20a), indicative of the formation of glycolic aldehyde intermediate. Therefore, we performed the comparative electrochemical and kinetic evaluation using EG, glycolic aldehyde, and glycolic acid as starting substrate, respectively. As shown in Supplementary Fig. 20b, c, the reaction rates of the three substrates to formate follows the order: glycolic aldehyde > EG > glycolic acid. These results indicate that glycolic acid is not the main intermediate for glycolic aldehyde transformation to formate. Combining our results and recent literature^23^, we propose that EG is initially oxidized to glycolic aldehyde, which is followed by rapid oxidative C–C cleavage to formate (Supplementary Fig. 20d). Meanwhile, minor glycolic aldehyde is oxidized to glycolic acid, which is followed by slow C–C cleavage to formate. After complete EG consumption, 90.2% yield of formate is obtained with good FE (82.5%) and carbon balance (>90%) (Fig. 2f). On the counterpart, water is reduced to hydrogen (Supplementary Fig. 20e).

**Fig. 3:**
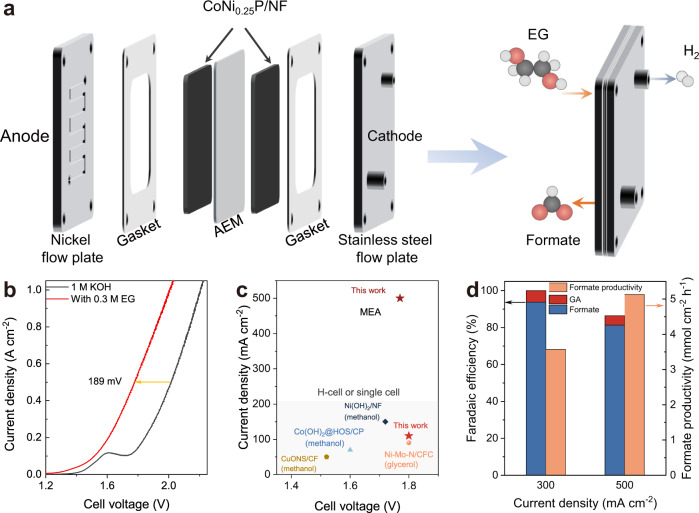
Membrane-electrode assembly (MEA) for EG oxidation. **a** The MEA setup for paired HER(−)//EG oxidation(+). **b** Polarization curves for water splitting and EG electrolysis in the MEA flow reactor at a scan rate of 10 mV s^−1^. **c** Current density for formate production as a function of cell voltage in literatures and this work. **d** Faradaic efficiency and productivity as function of current density for EG oxidation.

It is known that the Ohmic resistance would induce a huge energy loss at high current density, which represents one particular challenge in many electrolysis technologies^40,41^. To overcome this obstacle, a zero-gap membrane-electrode assembly (MEA) flow reactor is designed for converting EG into formate paired with H_2_ production using CoNi_0.25_P/NF as both the cathodic and anodic catalysts (Fig. 3a and Supplementary Fig. 21). The LSV polarization curves for traditional water splitting (HER//OER) and EG electrolysis (HER//EG oxidation) were acquired on this setup (Fig. 3b). The near-parallel polarization curves are presented for water splitting and EG electrolysis, showing ~189 mV decrease of cell voltage for reaching the same current density in the presence of EG in analyte, which in turn increases the cathodic energy efficiency. Compared with common H-cell or single cell for anodic formate production from alcohols (Supplementary Fig. 22 and Supplementary Table 5)^23,42–44^, the MEA reactor reaches a significantly higher current density (500 mA cm^−2^) at a low cell voltage (<1.8 V) (Fig. 3c). Moreover, the EG oxidation at a constant current density of 300 and 500 mA cm^−2^ in MEA give formate productivity of 3.6 and 5.1 mmol cm^−2^ h^−1^ (Fig. 3d), respectively, with good FE (>80%), representing one of the most advantageous systems (Supplementary Table 5).

### PET upcycling

**Fig. 4:**
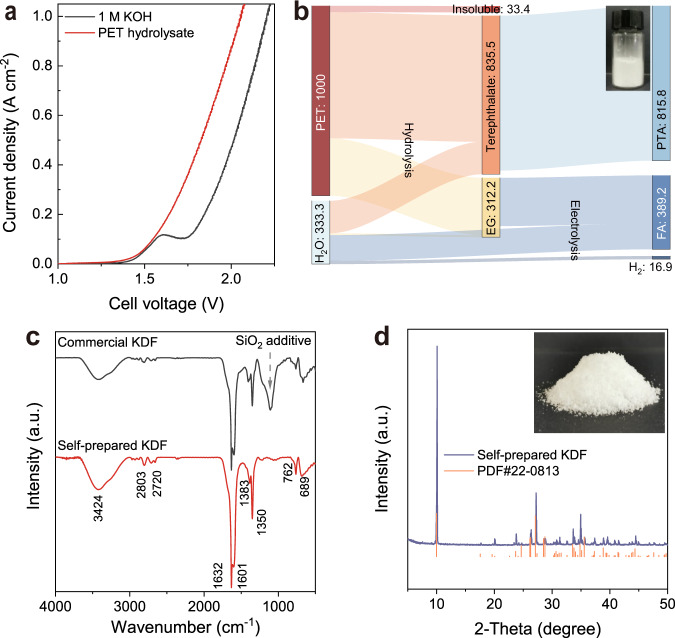
PET upcycling. **a** Polarization curves for electrolysis of PET hydrolysate. **b** Sankey diagram for the mass flow of PET upcycling. Photograph of separated high-purity PTA is shown (inset). **c** FT-IR spectra of self-prepared and commercial KDF. **d** XRD pattern and photograph (inset) of self-prepared KDF. a.u.: arbitrary units.

In the light of the above foundations, we aim to demonstrate the proof-of-concept production of KDF, PTA, and H_2_ from a real-world PET plastic. Initially, the polyester structure of PET was readily hydrolyzed into its monomers (PTA and EG) in an aqueous KOH solution at 60 °C with high yield (96.7%) (Supplementary Fig. 23). The PET hydrolysate was then used as the analyte for the MEA electrolyzer (Fig. 4a), in which EG was selectively oxidized to formate (Supplementary Fig. 24). After electrolysis, the PET hydrolysate was transformed into terephthalate and formate in the electrolyte. The PET electrolyte was then acidified by formic acid to regenerate pure PTA (Supplementary Figs. 25 and 26). As shown in the Sankey diagram of mass flow analysis (Fig. 4b), 1 kg of PET feedstock finally gave 389.2 g formate, 818.5 g PTA, and 16.9 g H_2_. After PTA separation, the filtrate stream contains formic acid and potassium formate, which was used for the synthesis of KDF through condensation and crystallization processes. Finally, 70% yield of white KDF crystal was obtained and confirmed by Fourier transformation infrared spectroscopy (FT-IR) and XRD (Fig. 4c, d).

In practical conditions, waste PET usually contain impurities, such as polyolefins^45^, poly(lactic acid) (PLA), and lipids^2^. The base inaccessible polyolefins can be removed from the hydrolysate during the pre-treatment by simple filtration (Supplementary Fig. 27a)^16^. Then, the lactate and glycerol from digested PLA and lipids can also be transformed to acetate and formate, respectively (Supplementary Fig. 27b, c). The acetic acid is also a common growth promoter for animals, which makes the process with some extent of compatibility for impure PET waste.

Although we demonstrated the HER//PET ER process in this work, replacing cathodic HER with other reductive organic transformations has the possibility to improve the profitability of PET reclaim. Particularly, the cost of formic acid production from electrocatalytic carbon dioxide reduction (CO_2_RR) is estimated to be ~180 $ ton^−1^, lower than the market price (400 $ ton^−1^)^46^. In this context, pairing CO_2_RR//PET ER is capable of increasing the revenues more than 200 $ ton^−1^ PET than that of HER//PET ER setup (Supplementary Fig. 28). However, more efforts are needed for this concept that is convergently transforming gaseous and solid wastes into valuable KDF, especially the reactor design.

### Catalyst stability and structural evolution

**Fig. 5:**
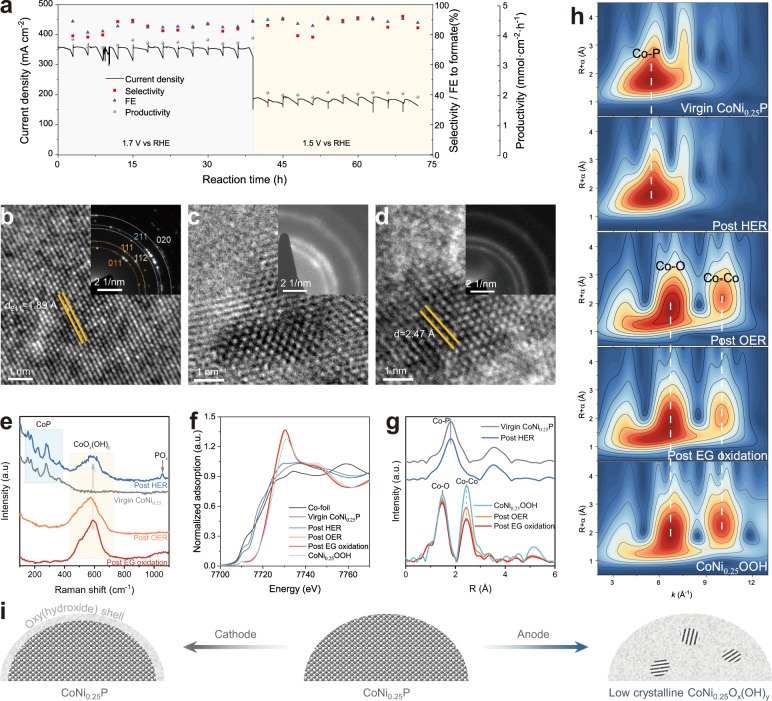
Catalytic stability and structural evolution of CoNi_0.25_P catalyst. **a** Chronoamperometric stability test for EG oxidation at 1.7 V vs RHE for 13 cycles and 1.5 V vs RHE for 11 cycles. The electrolyte was refreshed every 3 h. **b**–**d** TEM images and corresponding SAED patterns of CoNi_0.25_P after HER (**b**), OER (**c**), and EG oxidation (**d**) reaction for 1 h. **e** Raman spectra, **f** Co K-edge XANES profiles, **g** Co K-edge EXAFS spectra, and **h** Wavelet transforms for Co K-edge EXAFS spectra for virgin and spent CoNi_0.25_P. **i** Schematic illustration of the structural evolution of CoNi_0.25_P catalyst under reaction conditions.

The operating stability of CoNi_0.25_P/NF was evaluated at a constant potential of 1.7 V vs RHE for 39 h and 1.5 V vs RHE for 33 h in HER(−)//EG oxidation(+) system. As shown in Fig. 5a, CoNi_0.25_P/NF maintains a current of around 350 mA cm^−2^ at 1.7 V vs RHE and 180 mA cm^−2^ at 1.5 vs RHE with high FE and selectivity of formate (80–95%), indicating the high stability of CoNi_0.25_P/NF for EG oxidation and HER. It also exhibits excellent stability for water splitting at 1.7 V vs RHE for 60 h with a steady current density of ~159 mA cm^−2^ (Supplementary Fig. 29a).

It should be noted that an increase of current density (20–40 mA cm^−2^) in both water splitting and EG oxidation was observed in the initial period (<1 h) when using a virgin CoNi_0.25_P/NF catalyst (Supplementary Fig. 29a, b), suggesting a possible catalyst activation process. This might be a result of catalyst surface reconstruction, which has been identified in many electrocatalysts (especially for OER)^39,47,48^ and photocatalysts^49,50^. For better understanding of the reconstruction of the catalyst, a carbon cloth-supported CoNi_0.25_P catalyst was prepared (Supplementary Fig. 30) to eliminate the interference of NF matrix in characterization of materials after EG oxidation reactions as well as HER and OER at 1.7 V vs RHE. The combined results of TEM, SAED, XRD, and elemental analysis (Fig. 5b and Supplementary Fig. 31) reveal that the crystalline structure and composition of CoNi_0.25_P are well retained after HER. In contrast, the CoNi_0.25_P catalyst undergoes complete reconstruction after OER and EG oxidation reactions, and it transforms into a low crystalline metal oxy(hydroxide) analogue (Fig. 5c, d and Supplementary Figs. 32 and 33), similar to recently reported Co_9_S_8_ catalyst for alkaline OER^47^.

Moreover, the Raman spectrum of the spent CoNi_0.25_P catalyst for HER displays multiple peaks, including a collection of peaks at 130–400 cm^−1^ corresponding to CoP^51^, overlapped peaks at 410–720 cm^−1^ for CoO_*x*_(OH)_*y*_^52,53^, and a peak at 1056 cm^−1^ of PO_*x*_ (Fig. 5e). This can be explained by the oxidation and dissolution of transition metal phosphide in alkaline media and redeposition to form an amorphous metal oxy(hydroxide) shell structure over CoNi_0.25_P^33^. However, only the peaks of CoO_*x*_(OH)_*y*_ are observed in the spectra of the spent CoNi_0.25_P catalysts after OER and EG oxidation (Fig. 5e), which is consistent with the results of XRD, HRTEM, and elemental analysis (Supplementary Figs. 32 and 33).

We further investigated the electronic and geometric structures of the virgin and spent CoNi_0.25_P catalyst using X-ray absorption near-edge structure (XANES) and EXAFS. Both Co and Ni K-edge XANES spectra of the spent CoNi_0.25_P of HER slightly shift toward higher-energy region (Fig. 5f and Supplementary Fig. 34) compared with the virgin CoNi_0.25_P material, indicating a moderate oxidation of Co and Ni. The adsorption energy and white line intensity of Co XANES profiles for the spent CoNi_0.25_P catalysts of OER and EG oxidations are significantly increased and similar with the spectrum of CoNi_0.25_OOH reference, suggesting the oxidation state of Co are predominant in +3 valence. These results are also confirmed by XPS analysis (Supplementary Fig. 35). Moreover, the EXAFS and wavelet transform (WT) analysis of EXAFS spectra also verify the phenomenon that CoNi_0.25_P catalyst remains its main structure during HER, while it evolves into coordination unsaturated metal oxy(hydroxide) analogue during anodic EG oxidation (Fig. 5g, h).

As illustrated in Fig. 5i, the starting CoNi_0.25_P material evolves into a CoNi_0.25_P/CoNi_0.25_O_*x*_(OH)_*y*_ core–shell structure at cathode, while it is drastically oxidized and reconstructed into low-crystalline CoNi_0.25_O_*x*_(OH)_*y*_ at anode. The EXAFS spectra at different reaction times reveal that the reconstruction of virgin CoNi_0.25_P is completed within 1 h and stabilized in the following running cycles (Supplementary Fig. 36). This structural evolution might account for the catalyst activation process and the advantageous performance of CoNi_0.25_P compared with the crystalline CoNi_0.25_OOH for EG oxidation^39^.

## Discussion

We demonstrate an electrocatalytic upcycling strategy for PET waste to produce valuable H_2_, PTA, and KDF. Preliminary TEA highlights the economic possibility of this process at commercially relevant current densities (>100 mA cm^−2^). An optimized CoNi_0.25_P/NF material was synthesized that achieves high current densities of ~350 mA cm^−2^ at 1.7 V vs RHE in an H-cell and 500 mA cm^−2^ at ~1.8 V cell voltage in a MEA, with high formate selectivity (>80%) and FE (>80%). The advantageous performance for EG oxidation can be attributed to the structural evolution of CoNi_0.25_P catalyst toward low-crystalline CoNi oxyhydroxide. This work may open a route for profitable and sustainable preparation of value-added commodity chemicals and clean H_2_ fuel from plastic waste.

## Methods

### Synthesis of catalysts

First, the NF (3 × 4 cm) pieces were washed with ethanol, 0.5 M HCl, and deionized water, which were used as both work and counter electrodes for electrodeposition in a three-electrode system with a saturated calomel reference electrode (SCE). Typically, the CoNi_0.25_(OH)_2_/NF was prepared in an aqueous solution with 100 mM Co(NO_3_)_2_ and 25 mM Ni(NO_3_)_2_ by applying a constant current of −80 mA for 300 s. Then, the CoNi_0.25_(OH)_2_/NF material was phosphorated at 300 °C in argon atmosphere for 2 h using NaH_2_PO_2_ as phosphorus source to obtain CoNi_0.25_P/NF. The loading of metal hydroxide was calculated to be 2.85 ± 0.13 mg cm^−2^. The other electrodes were also prepared by similar procedures with varying concentration of Ni precursor (Supplementary Table 4).

### Material characterizations

Scanning electron microscope (SEM) images of materials were acquired on Zeiss Supra 55. Transmission electron microscope (TEM) images and selected area electron diffraction (SAED) pattern were acquired on FEI TECNAI G^2^ at 200 keV. X-ray diffraction (XRD) patterns were recorded on a Bruker D8 Advance diffractometer at 40 mA and 40 kV using Cu Kα radiation. X-ray photoelectron spectroscopy (XPS) was performed on a Kratos Axis Supra using monochromatic Al Kalph source (150 W). Metal contents in catalysts were determined by ICP-AES on a Thermo ICAP6300 Radial.

### Electrochemical evaluation

All electrochemical experiments were performed in an H-type cell separated by anion exchange membrane (Sustainion^®^ 37-50, Dioxide Materials) on an electrochemical workstation (CHI 760E, CH Instruments, Inc.). The powder catalysts (20% Pt/C (Johnson Matthey) and RuO_2_ (Alfa Aesar)) were dispersed in aqueous isopropanol solution with Nafion by sonification and sprayed on carbon fiber paper (CFP) with a mass loading of 1 ± 0.1 mg cm^−2^. The geometric area of self-supported catalysts and CFP-supported catalysts is 1 cm × 1 cm for all experiments. The tests were carried out in a three-electrode system, using saturated Ag/AgCl and platinum foil as reference and counter electrodes, respectively. All potentials measured against Ag/AgCl were converted to the reversible hydrogen electrode (RHE) scale using *E*_RHE_ = *E*_Ag/AgCl_ + 0.197 + 0.059 pH. Solution resistance was determined by potentiostatic electrochemical impedance spectroscopy and manually compensated for polarization curves.

The EG oxidation reactions were carried out at potentiostatic mode without iR compensation. After reactions, the concentration of substrate and products were quantified by HPLC (Angilent 1260) equipped with organic acid column (Coregel 87H3) using 5 mM aqueous H_2_SO_4_ as mobile phase. The Faradaic efficiency (FE), formate selectivity, and productivity were calculated with the following equations:

$${{{{{\rm{FE}}}}}}( \% )=100 \% \times \frac{{{{{{\rm{mole}}}}}}\,{{{{{\rm{of}}}}}}\,{{{{{\rm{produced}}}}}}\,{{{{{\rm{product}}}}}}}{{{{{{\rm{total}}}}}}\,{{{{{\rm{charge}}}}}}\,{{{{{\rm{passed}}}}}}/(n\times 96485\,{{{{{\rm{C}}}}}}\,{{{{{{\rm{mol}}}}}}}^{-1})}$$

$${{{{{\rm{Formate}}}}}}\,{{{{{\rm{selectivity}}}}}}\,( \% )=100 \% \times \frac{{{{{{\rm{mole}}}}}}\,{{{{{\rm{of}}}}}}\,{{{{{\rm{produced}}}}}}\,{{{{{\rm{formate}}}}}}/2}{{{{{{\rm{mole}}}}}}\,{{{{{\rm{of}}}}}}\,{{{{{\rm{converted}}}}}}\,{{{{{\rm{EG}}}}}}}$$

$${{{{{\rm{Formate}}}}}}\,{{{{{\rm{productivity}}}}}}\;({{{{{\rm{mmol}}}}}}\,{{{{{{\rm{cm}}}}}}}^{-2}\,{{{{{{\rm{h}}}}}}}^{-1})=\frac{{{{{{\rm{amount}}}}}}\,{{{{{\rm{of}}}}}}\,{{{{{\rm{produced}}}}}}\,{{{{{\rm{formate}}}}}}\,({{{{{\rm{mmol}}}}}}\,)}{{{{{{\rm{area}}}}}}\,{{{{{\rm{of}}}}}}\,{{{{{\rm{anode}}}}}}\,({{{{{{\rm{cm}}}}}}}^{2})\times {{{{{\rm{reaction}}}}}}\,{{{{{\rm{time}}}}}}\,({{{{{\rm{h}}}}}})}$$

where *n* is the number of electron transfer for each product formation, *n* = 3 for formate, *n* = 4 for glycollate; 96485 C mol^−1^ is the Faraday constant.

The configuration of membrane-electrode assembly (MEA) electrolyzer was depicted in Supplementary Fig. 21 and composed of metal housings, gaskets, as-prepared catalysts, and AEM. The electrochemical performance testing of the MEA electrolyzer was performed on an electrochemical workstation with a power amplifier (CS1005, CorrTest).

### Hydrolysis of PET

For depolymerization of PET, 6.3 g dried powder (PET^TM^CB-102, 300 mesh, Dupont) was dispersed in 100 mL of 2 M KOH aqueous solution in a flask. Then, the flask was sealed with a rubber stopper and heated on a hotplate at 60 °C with stirring (1500 rpm) for specific time (3, 6, 12, 18 h). After reactions, the yields of EG and PTA in PET hydrolysate were determined by HPLC.

### Products separation from PET electrolyte

The obtained PET hydrolysate was electrolyzed in MEA electrolyzer at 1.7 V for converting EG into formate. After reaction, anhydrous formic acid was added into PET electrolyte to adjust the pH to 3. Meanwhile, white PTA was precipitated from the electrolyte, which was subsequently filtered by membrane (0.22 µm) and washed with deionized water. The filtrate containing formate and formic acid were concentrated with rotary evaporation (−0.1 MPa, 50 °C) to remove water. The hot oversaturated solution was immediately filtered with a filter paper and cooled at 4 °C for the crystallization of potassium diformate (KDF). Finally, the as-obtained PTA and KDF were dried in vacuum oven at 60 °C.

## Supplementary information

Supplementary information

## Data Availability

Additional data related to this study are available from the corresponding authors on reasonable request. Source data are provided with this paper.

## Source data

Source data.
