# Supplementary material for: Electrocatalytic upcycling of polyethylene terephthalate to commodity chemicals and H2 fuel
Source: Nat Commun. 2021 Aug 17;12:4679. doi: 10.1038/s41467-021-25048-x (PMC8371182; doi:10.1038/s41467-021-25048-x)
Supplement: Supplementary file 1 — Supplementary information [file 41467_2021_25048_MOESM1_ESM.pdf]

Supplementary Information

Electrocatalytic Upcycling of Polyethylene Terephthalate to Commodity Chemicals and H_2 fuel

Hua Zhou[1,4], Yue Ren[2,4], Zhenhua Li[2,4], Ming Xu[2], Ye Wang[1], Ruixiang Ge[1], Xianggui Kong[2], Lirong Zheng[3], Haohong Duan*[1]

[1]Department of Chemistry, Tsinghua University, Beijing 100084, China

[2] State Key Laboratory of Chemical Resource Engineering, College of Chemistry, Beijing University of Chemical Technology, Beijing 100029, China

[3]Institute of High Energy Physics, the Chinese Academy of Sciences, Beijing 100049, China

[4] These authors contributed equally: Hua Zhou, Yue Ren, Zhenhua Li

*Corresponding authors. E−mail addresses: hhduan@mail.tsinghua.edu.cn (H. Duan)

Supplementary Figures

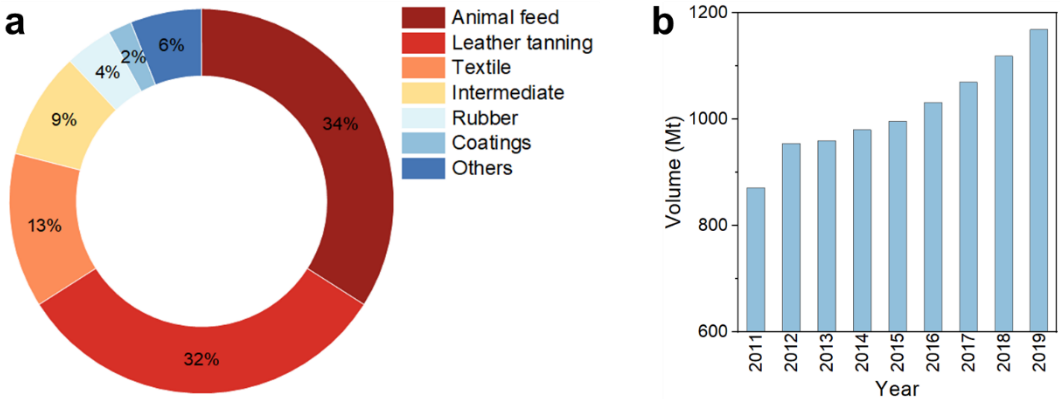

Supplementary Figure 1 Trends of formic acid market. a Application field of formic acid. The data are obtained from online, https://www.oxfa.eu/en/markets/. **b** The increasing demand of formic acid for feed additives. The data are obtained from Mordor Intelligence. https://www.mordorintelligence.com/industry-reports/formic-acid-market.

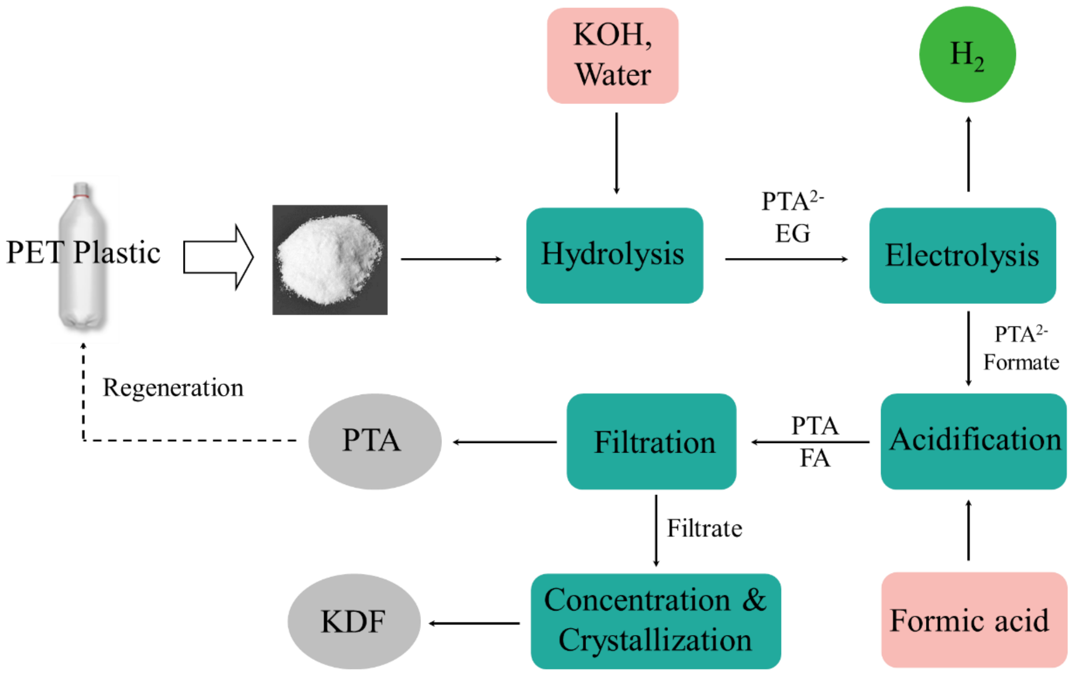

Supplementary Figure 2 Schematic operating units of the integrated process for PET upcycling (Route I). The targeted products are PTA, H_2, and KDF.

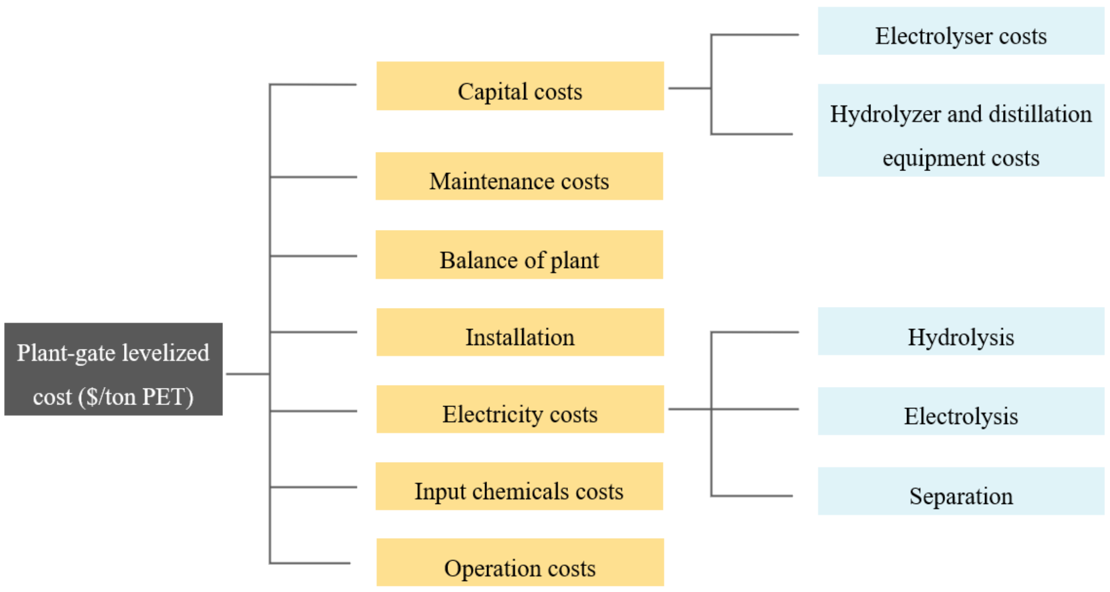

Supplementary Figure 3 Model used for calculating the plant-gate levelized cost of the process.

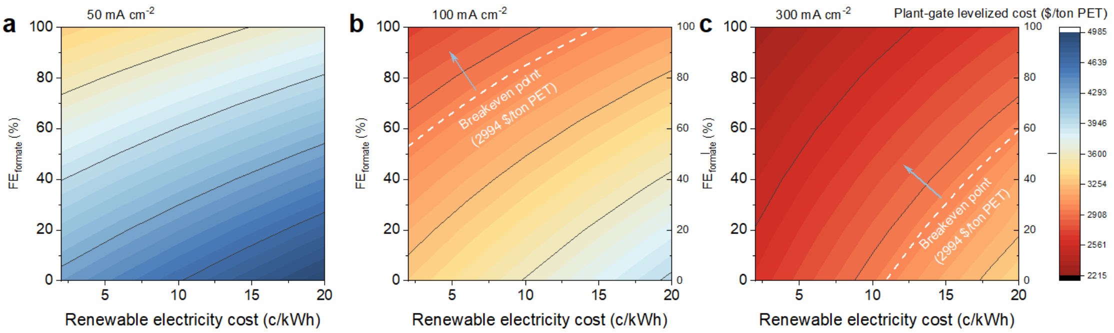

Supplementary Figure 4 Plant-gate levelized cost for processing PET as function of Faradaic efficiency to formate ($FE_{formate}$), renewable electricity cost, and current density. a 50 mA cm^{-2}, **b** 100 mA cm^{-2}, **c** 300 mA cm^{-2}.

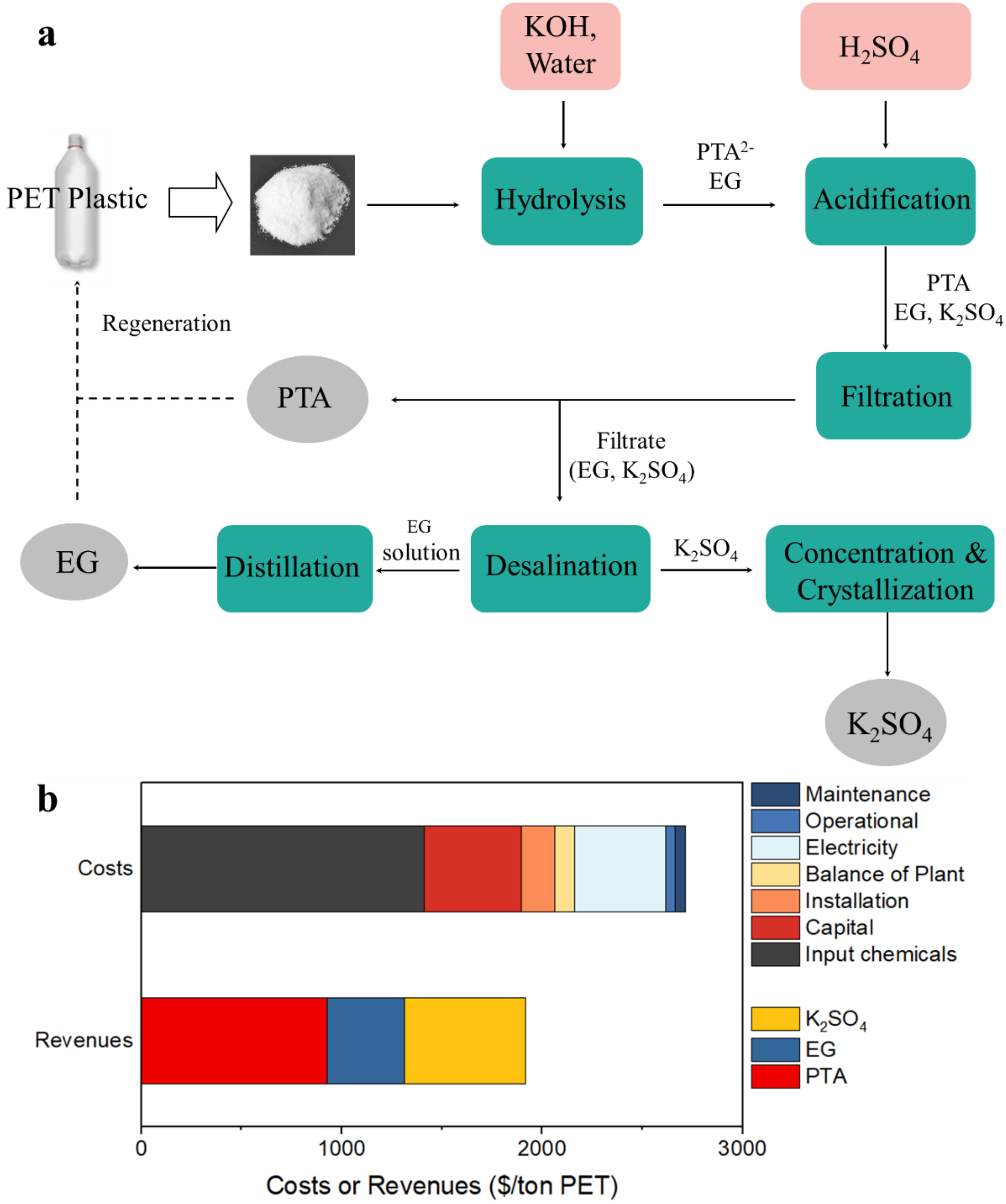

Supplementary Figure 5 Route II for PTA and EG recycling. a Schematic illustration of the process, the final products are K_2SO_4, EG, and PTA. **b** TEA analysis of this process. Assuming 90% of K_2SO_4 and 100% of EG are recycled.

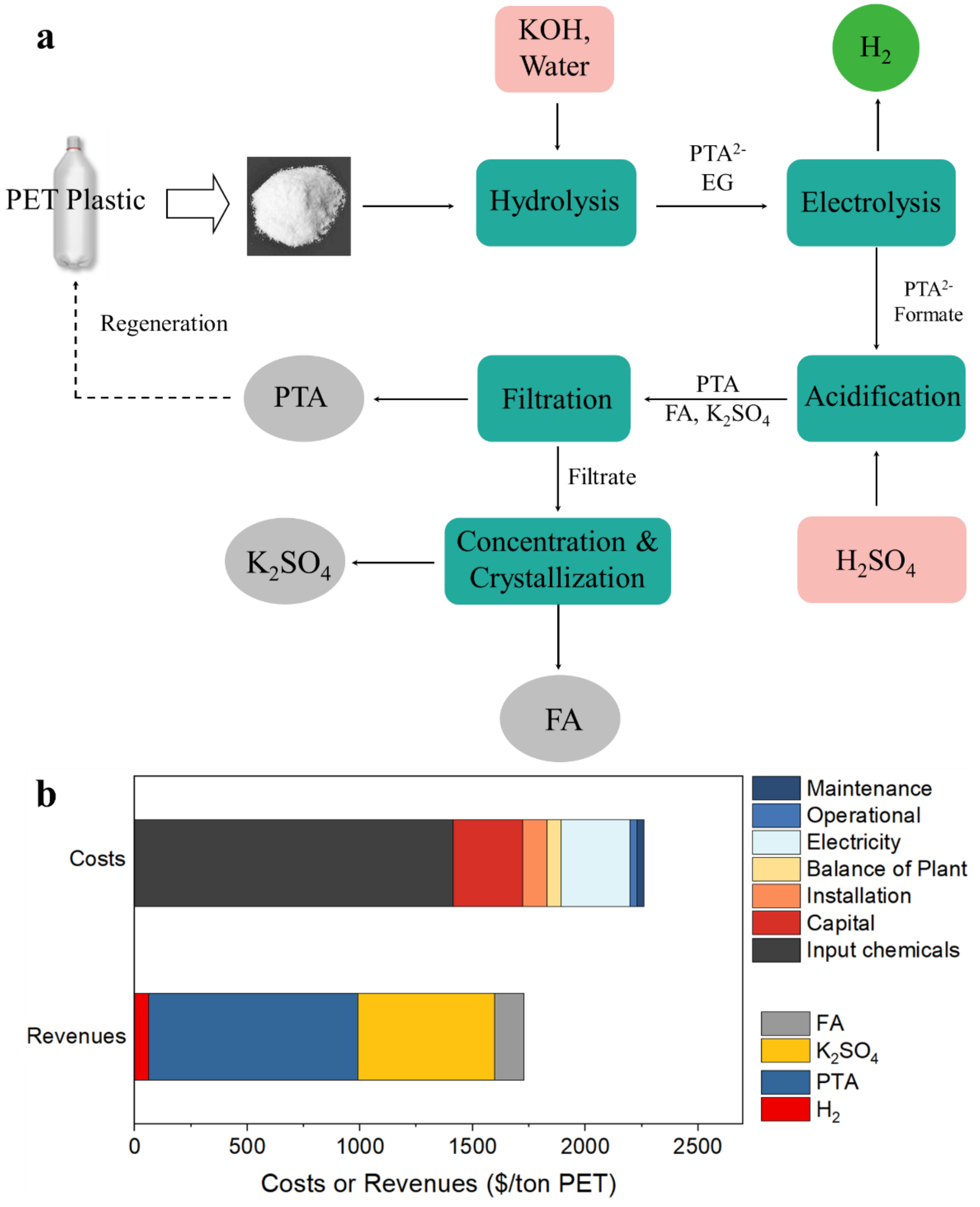

Supplementary Figure 6 Route III for PET reclaim. a Schematic illustration of the process, the final products are FA, K_2SO_4, PTA, and H_2. **b** TEA analysis of this process. Assuming 90% of K_2SO_4 and 100% of FA are recycled.

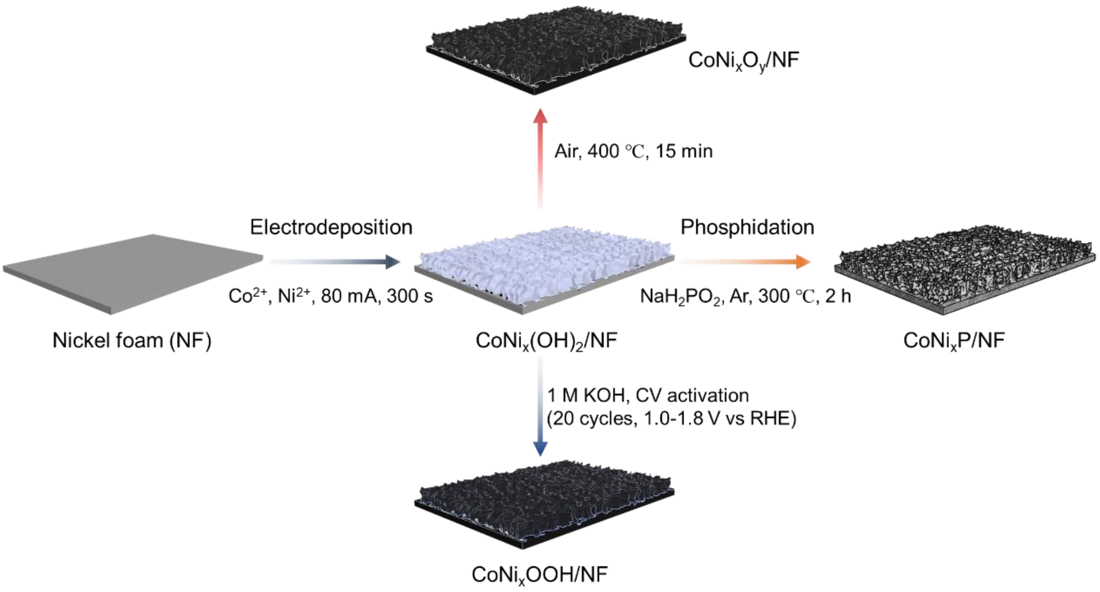

Supplementary Figure 7 Schematic illustration of the preparation of $CoNi_xP$/NF and $CoNi_xOOH$/NF.

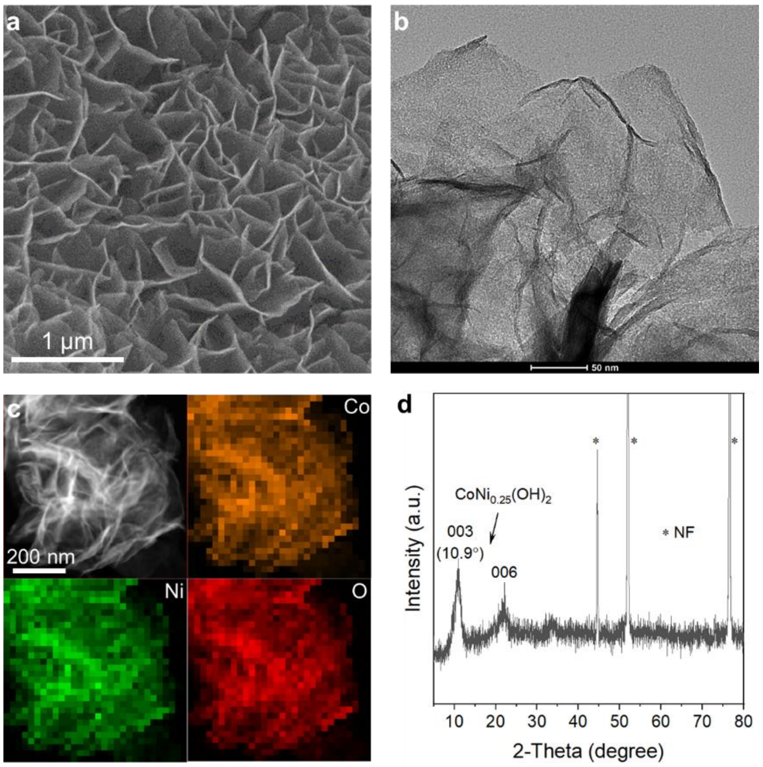

Supplementary Figure 8 Characterization of $CoNi_{0.25}(OH)_2$/NF material. **a** SEM image of $CoNi_{0.25}(OH)_2$ nanoarray on NF. **b** TEM image of $CoNi_{0.25}(OH)_2$ nanosheet. **c** Elemental mapping of $CoNi_{0.25}(OH)_2$ nanosheet. **d** XRD pattern of $CoNi_{0.25}(OH)_2$/NF. a.u.: arbitrary units.

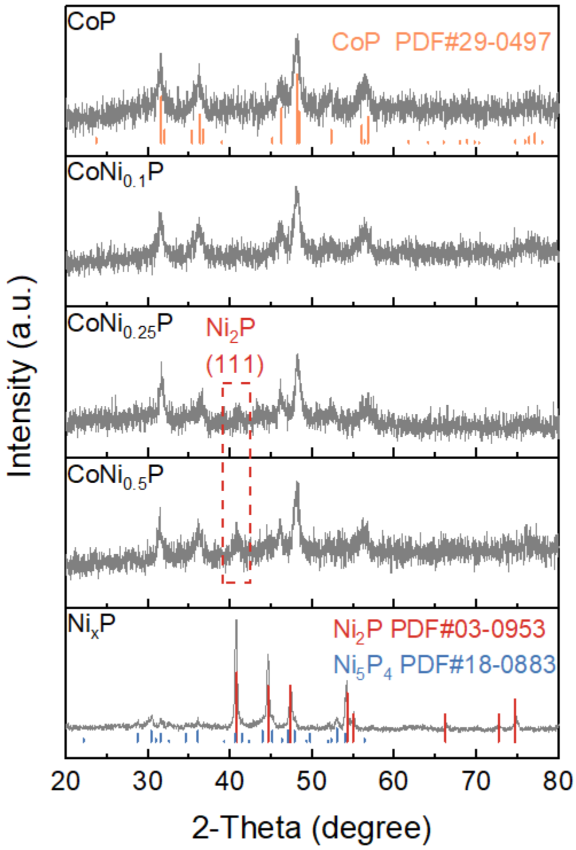

Supplementary Figure 9 XRD patterns of $CoNi_xP$ and Ni_2P.

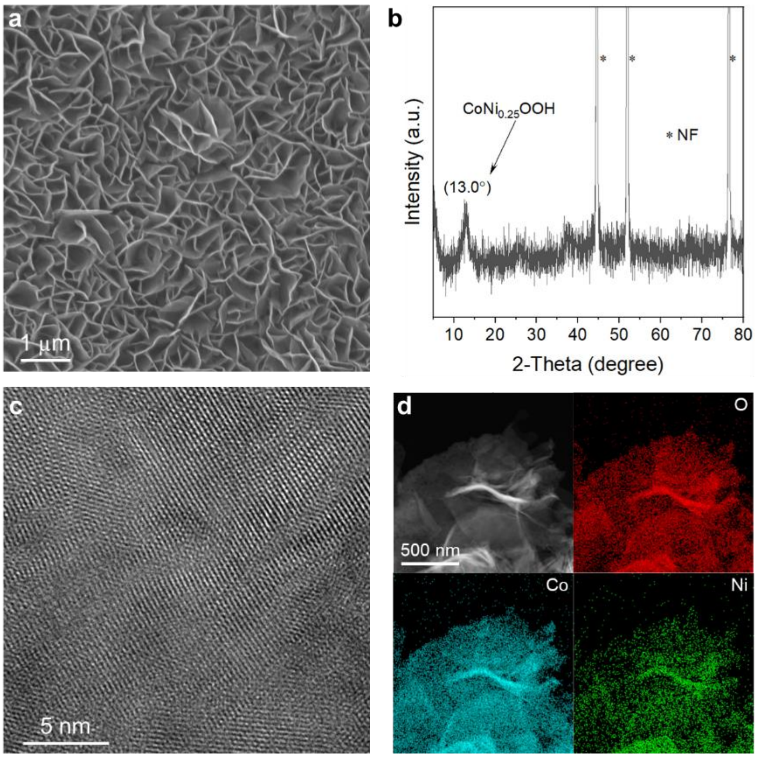

Supplementary Figure 10 Characterization of crystalline $CoNi_{0.25}OOH$/NF material. a SEM image of $CoNi_{0.25}OOH$ nanoarray on NF. **b** XRD pattern of $CoNi_{0.25}OOH$/NF. **c** HR-TEM image of

$CoNi_{0.25}OOH$ nanosheet. **d** Elemental mapping of $CoNi_{0.25}OOH$ nanosheet.

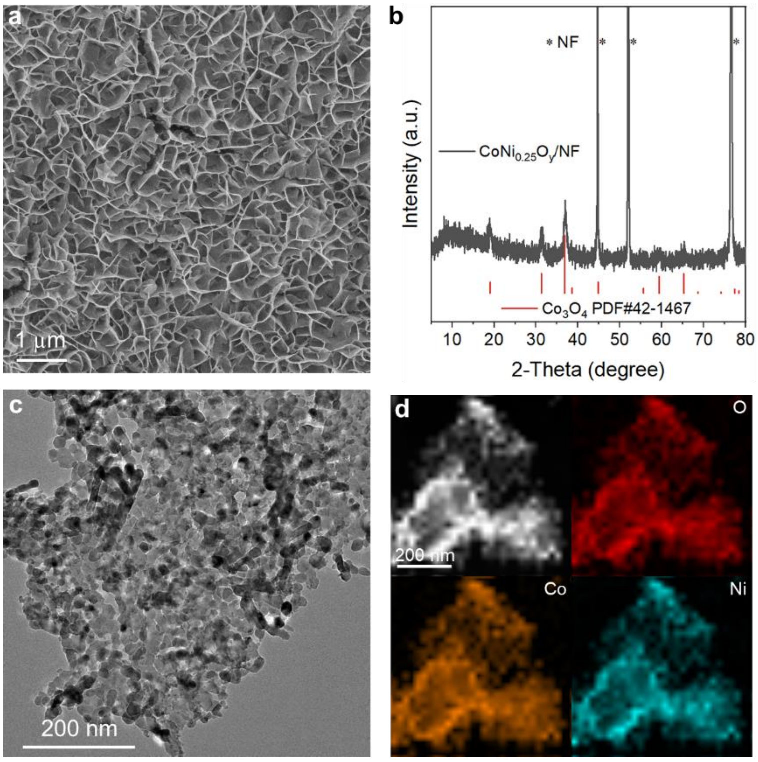

Supplementary Figure 11 Characterization of $CoNi_{0.25}O_y$/NF material. a SEM image of $CoNi_{0.25}O_y$ nanoarray on NF. **b** XRD pattern of $CoNi_{0.25}O_y$/NF. **c** TEM image of $CoNi_{0.25}O_y$ nanosheet. **d** Elemental mapping of $CoNi_{0.25}O_y$ nanosheet.

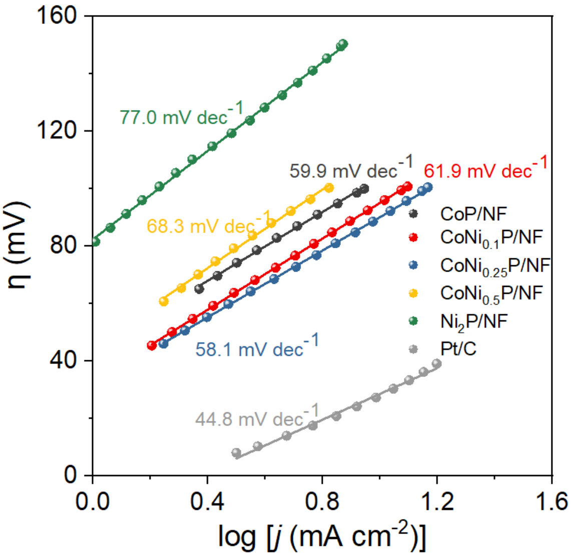

Supplementary Figure 12 Tafel plots derived from HER polarization curves.

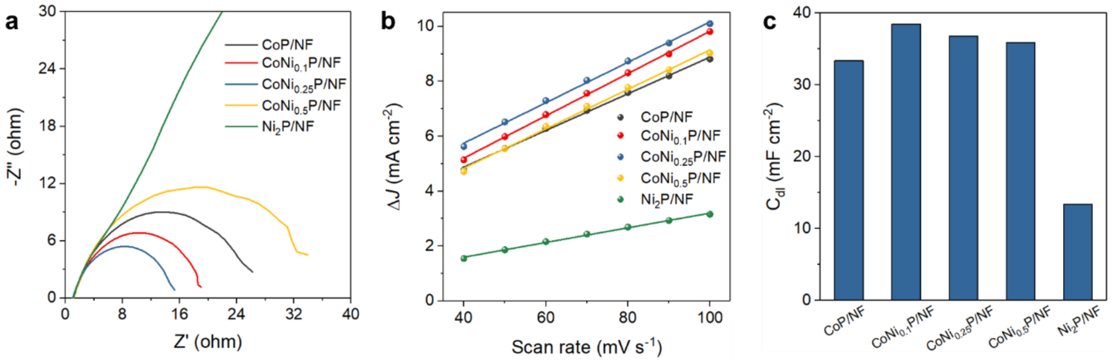

Supplementary Figure 13 a Electrochemical impedance spectra (EIS) of $CoNi_xP$/NF and Ni_2P/NF catalysts at a potential of -1.07 V vs RHE. **b** Charging current density differences (ΔJ = Ja−Jc) plotted against scan rates. The linear slope is equivalent to twice of the double-layer capacitance (C_{dl}). **c** C_{dl} of $CoNi_xP$/NF and Ni_2P/NF catalysts.

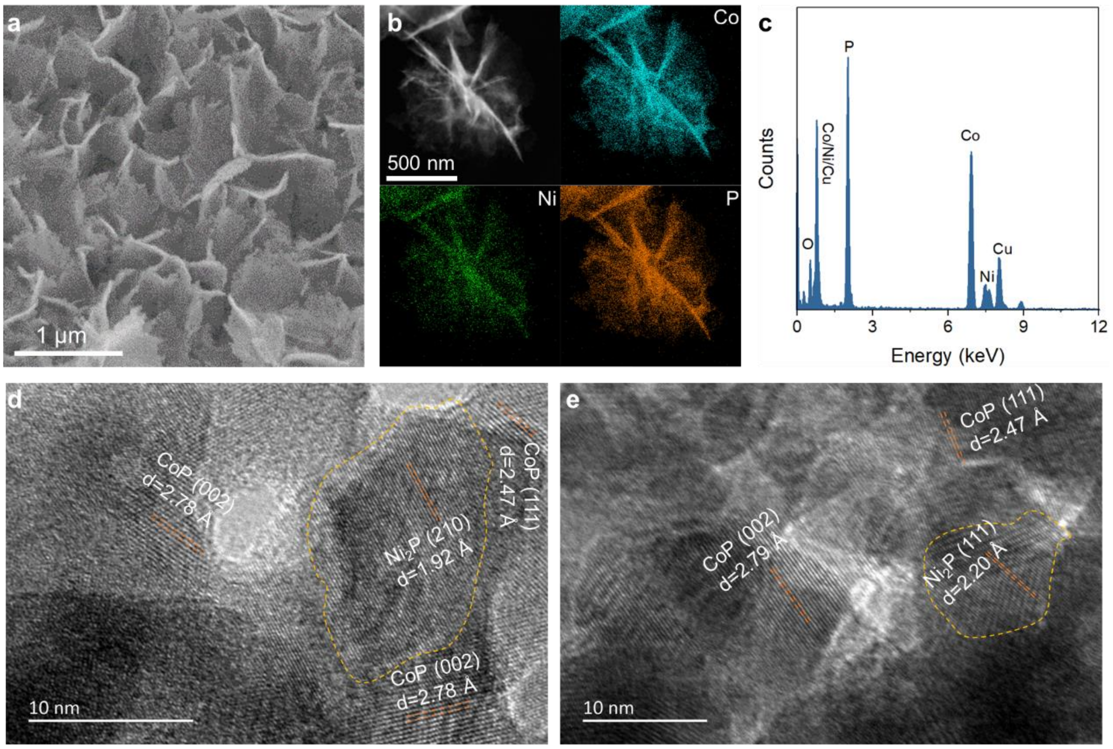

Supplementary Figure 14 Characterization of $CoNi_{0.25}P$/NF material. **a** SEM image of $CoNi_{0.25}P$ nanoarray on NF. **b** Elemental mapping of $CoNi_{0.25}P$ nanosheet. **c** EDS spectrum, **d**, **e** HRTEM images of $CoNi_{0.25}P$ nanosheet at different regions.

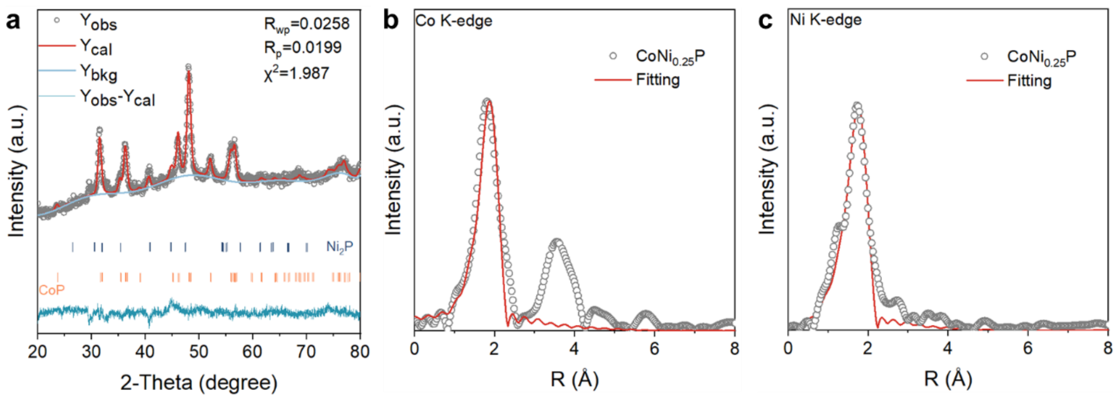

Supplementary Figure 15 a XRD Rietveld refinement and EXAFS fitting of **b** Co K-edge and **c** Ni K-edge of $CoNi_{0.25}P$ material.

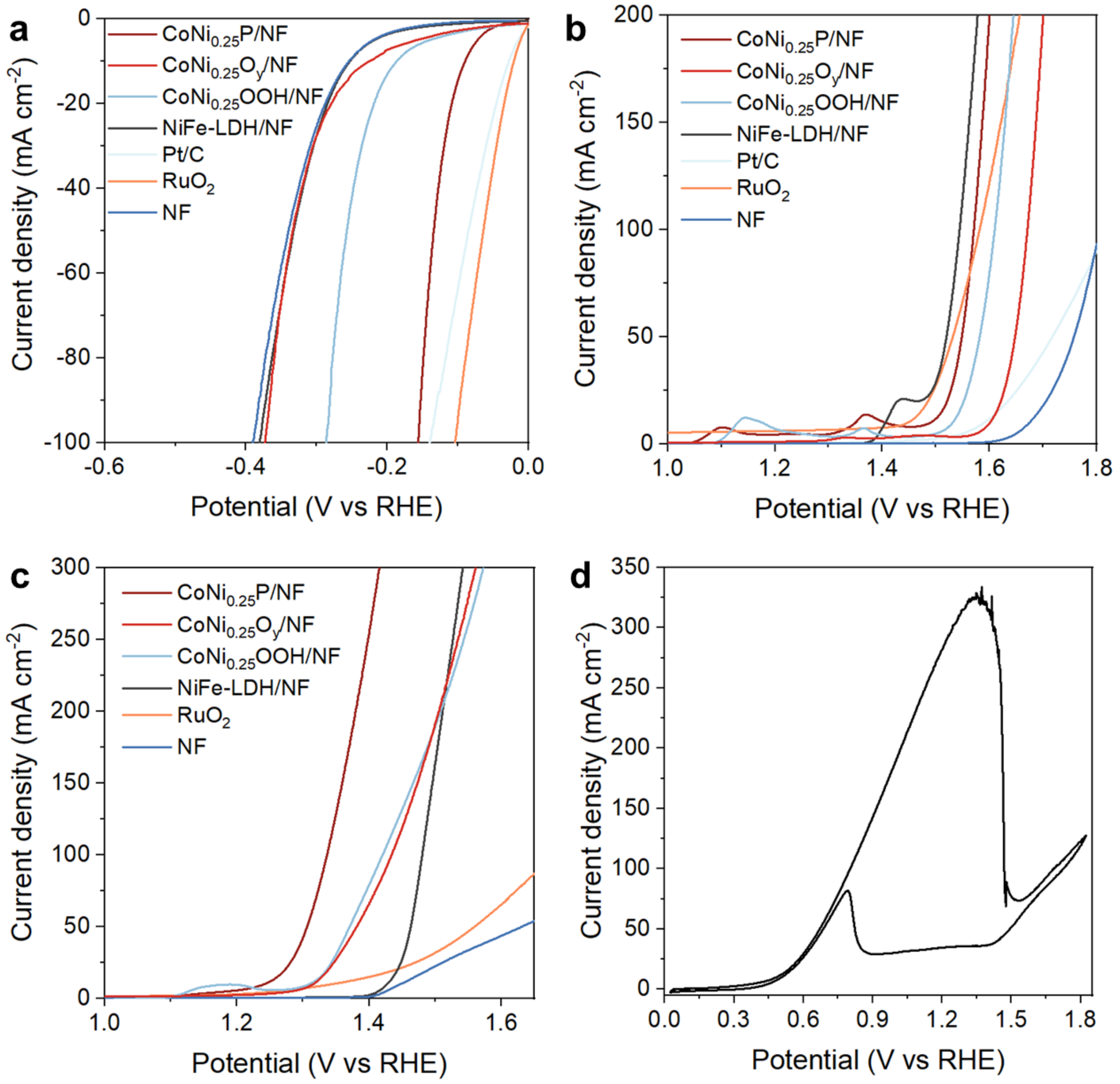

Supplementary Figure 16 Comparison of the catalytic activities of $CoNi_{0.25}P$/NF and common electrocatalysts. **a** LSV curves of catalysts for HER. **b** LSV curves of catalysts for OER. **c** LSV curves of catalysts for EG oxidation. **d** CV polarization curve of Pt/C for EG oxidation.

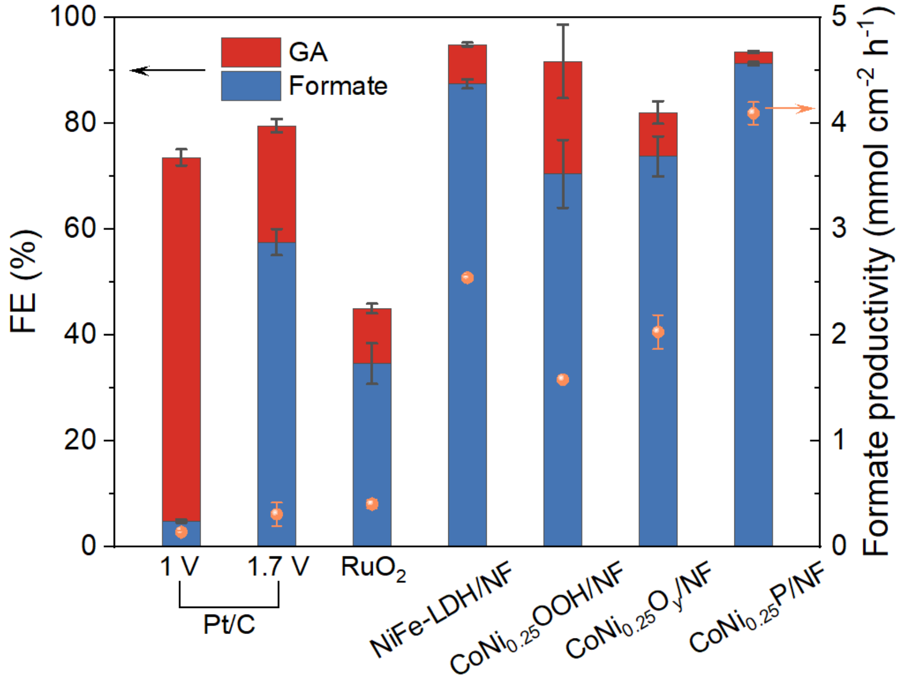

Supplementary Figure 17 FE of EG oxidation over various catalysts. Reaction conditions: 0.3 M EG in 1 M KOH electrolyte at 1.7 V vs RHE for 0.5 h over RuO_2, NiFe-LDH/NF, $CoNi_{0.25}OOH$/NF, $CoNi_{0.25}O_y$/NF, and $CoNi_{0.25}P$/NF. Error bars correspond to the standard deviation of three measurements.

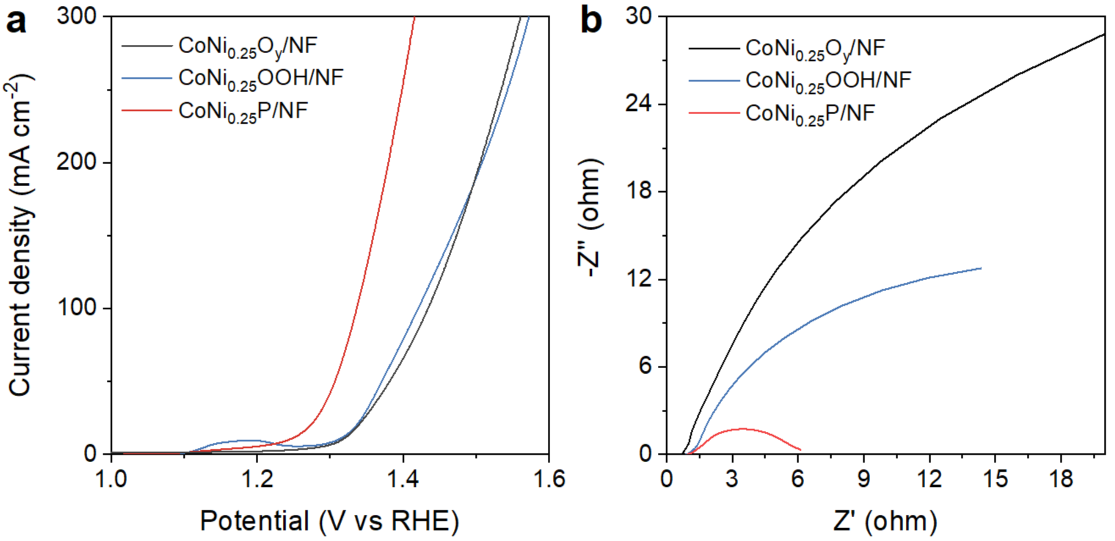

Supplementary Figure 18 Electrochemical evaluation of $CoNi_{0.25}(OH)_2$/NF derived phosphide, oxy(hydroxide), and oxide for EG oxidation. a LSV curves (85% iR corrected) of materials in 1M KOH with 0.3 M EG. **b** EIS of materials for EG oxidation at a potential of 1.25 V vs RHE.

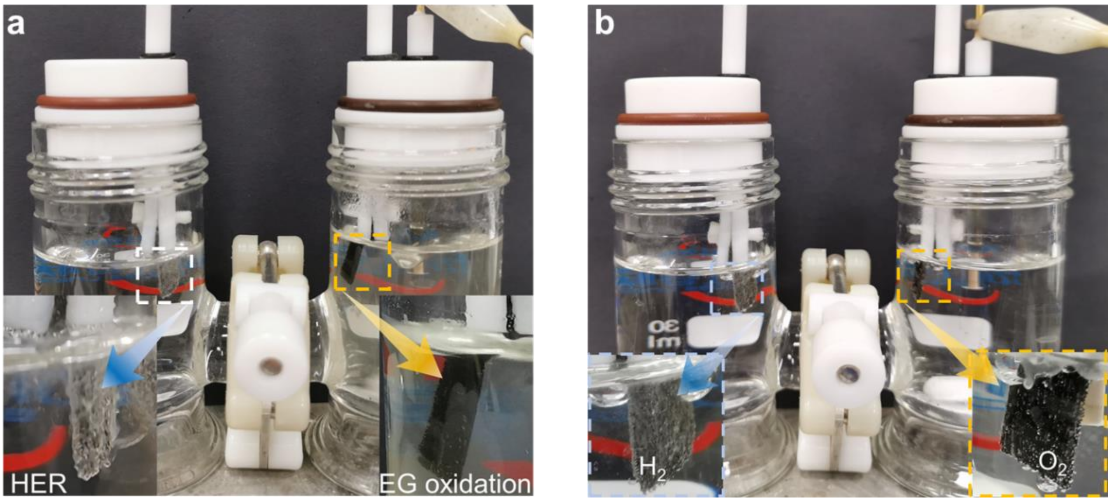

Supplementary Figure 19 a Photograph of H-cell configuration for HER//EG oxidation, showing the evolution of H_2 over $CoNi_{0.25}P/NF$. **b** Photograph of H-cell configuration for HER//OER, showing the evolution of H_2 and O_2 bubbles over $CoNi_{0.25}P/NF$ at cathode and anode, respectively.

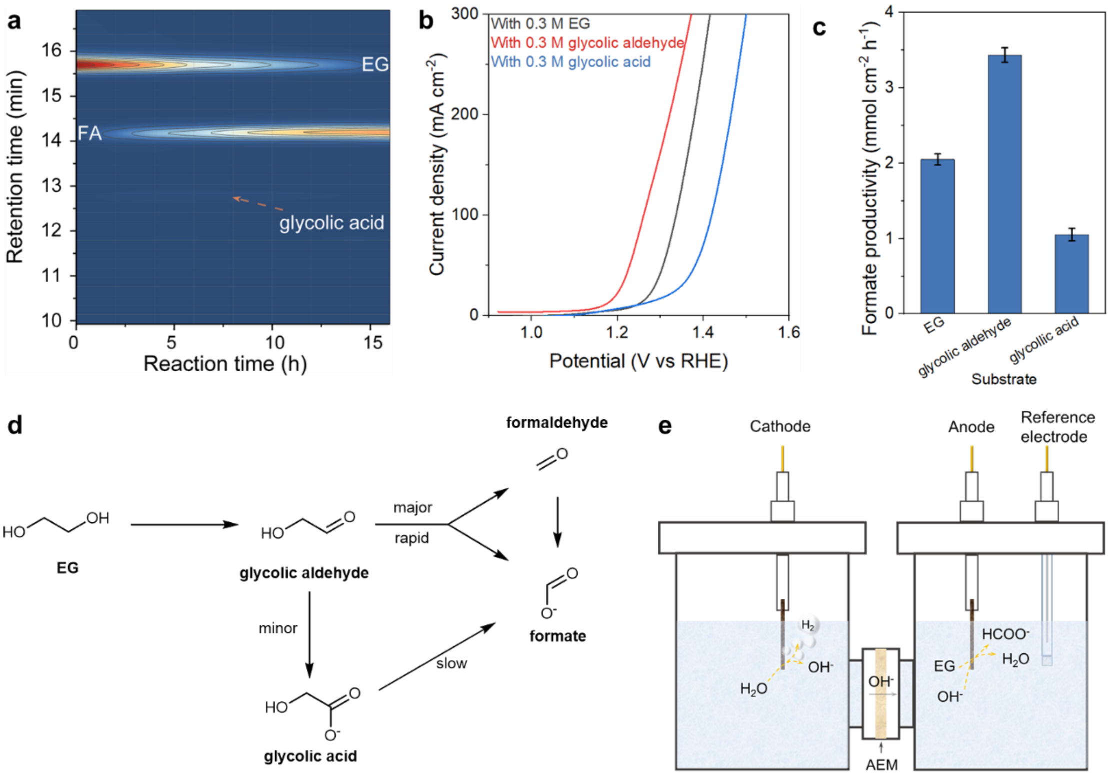

Supplementary Figure 20 a HPLC chromatograms of EG oxidation as function of reaction time. Reaction conditions: 0.3 M EG in 1 M KOH electrolyte at 1.5 V vs RHE over $CoNi_{0.25}P/NF$ anode. **b** LSV curves (85% iR corrected) for organic oxidation in 1 M KOH with 0.3 M substrate. **c** Formate

productivity at constant potential (1.5 V vs RHE) for different substrate. **d** Proposed reaction route for electrocatalytic EG oxidation. **e** The schematic illustration of whole electrolysis reaction.

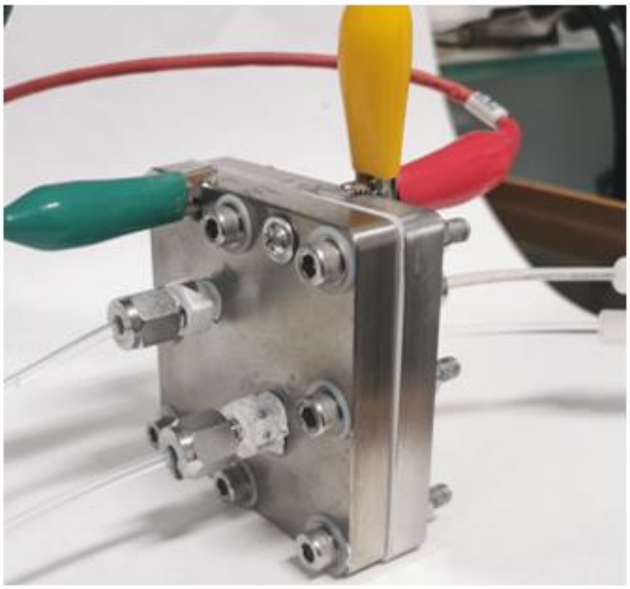

Supplementary Figure 21 Photograph of MEA for experiments.

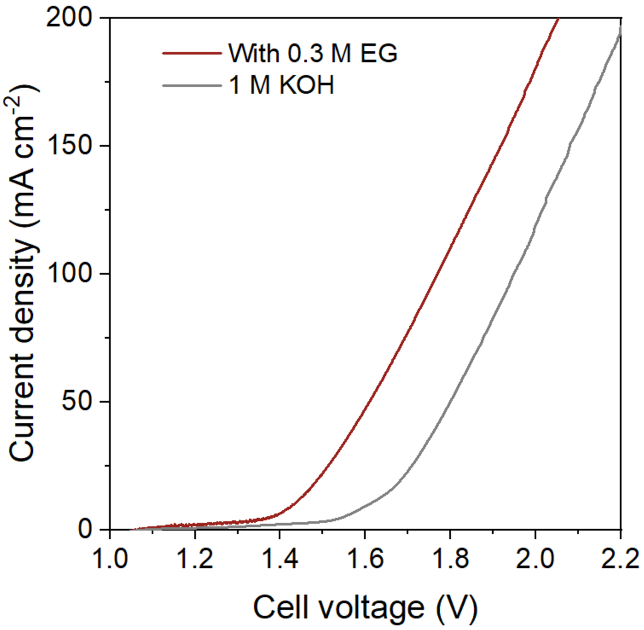

Supplementary Figure 22 LSV curves for OER and EG oxidation in single cell.

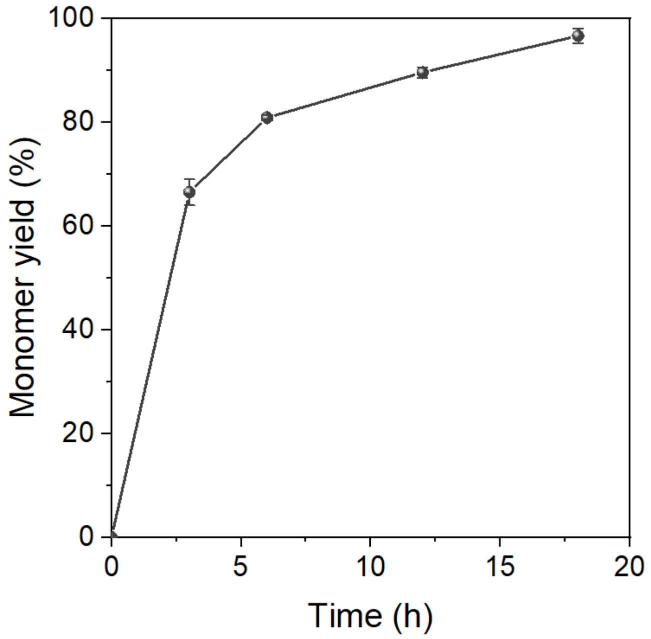

Supplementary Figure 23 Monomer yield of PET hydrolysis as the function of time, 0.3 M PET in 2 M KOH at 60 °C.

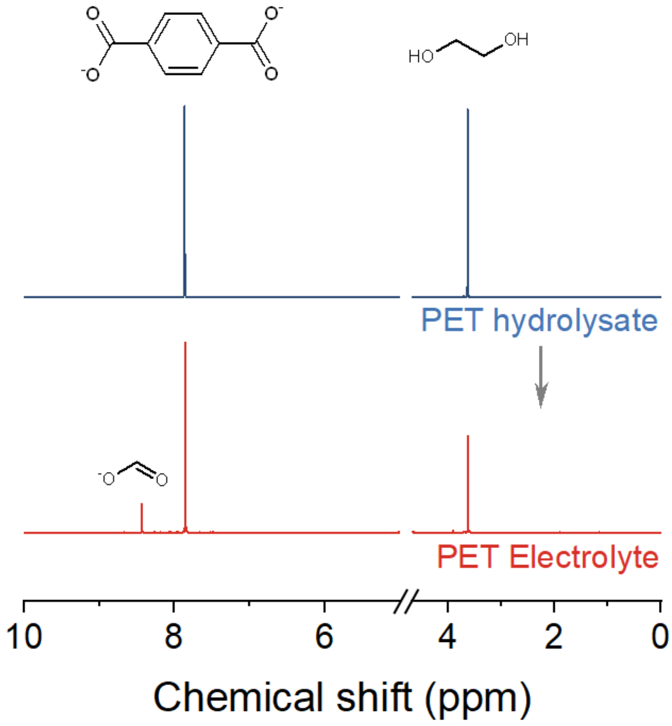

Supplementary Figure 24 ^{1}H NMR spectra of PET hydrolysate and electrolyte.

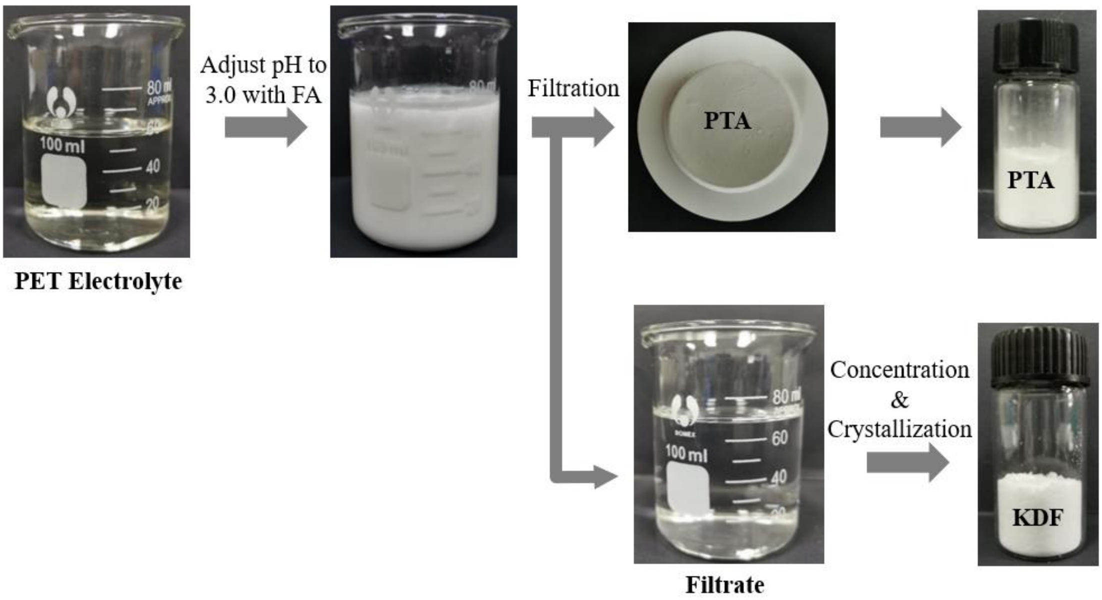

Supplementary Figure 25 Photo pictures for demonstrating procedures of products separation from PET electrolyte.

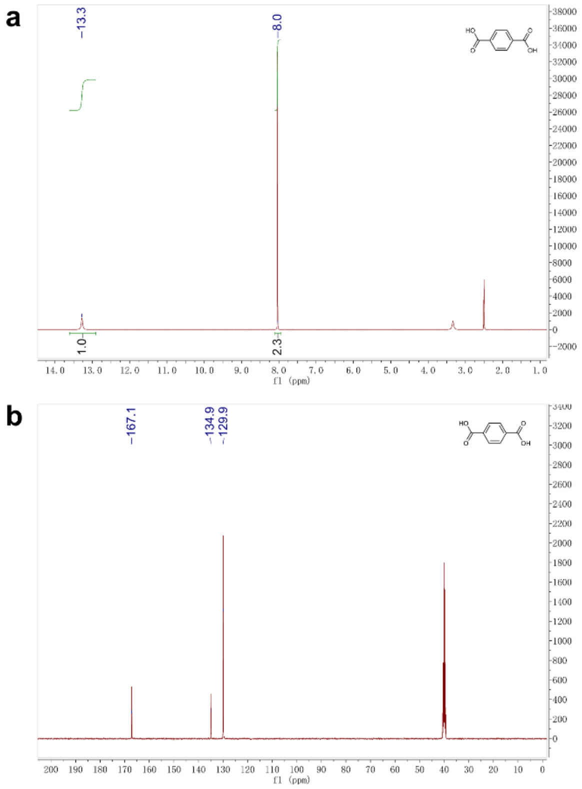

Supplementary Figure 26 NMR for regenerated PTA. **a** ^{1}H NMR spectrum. **b** ^{13}C NMR spectrum.

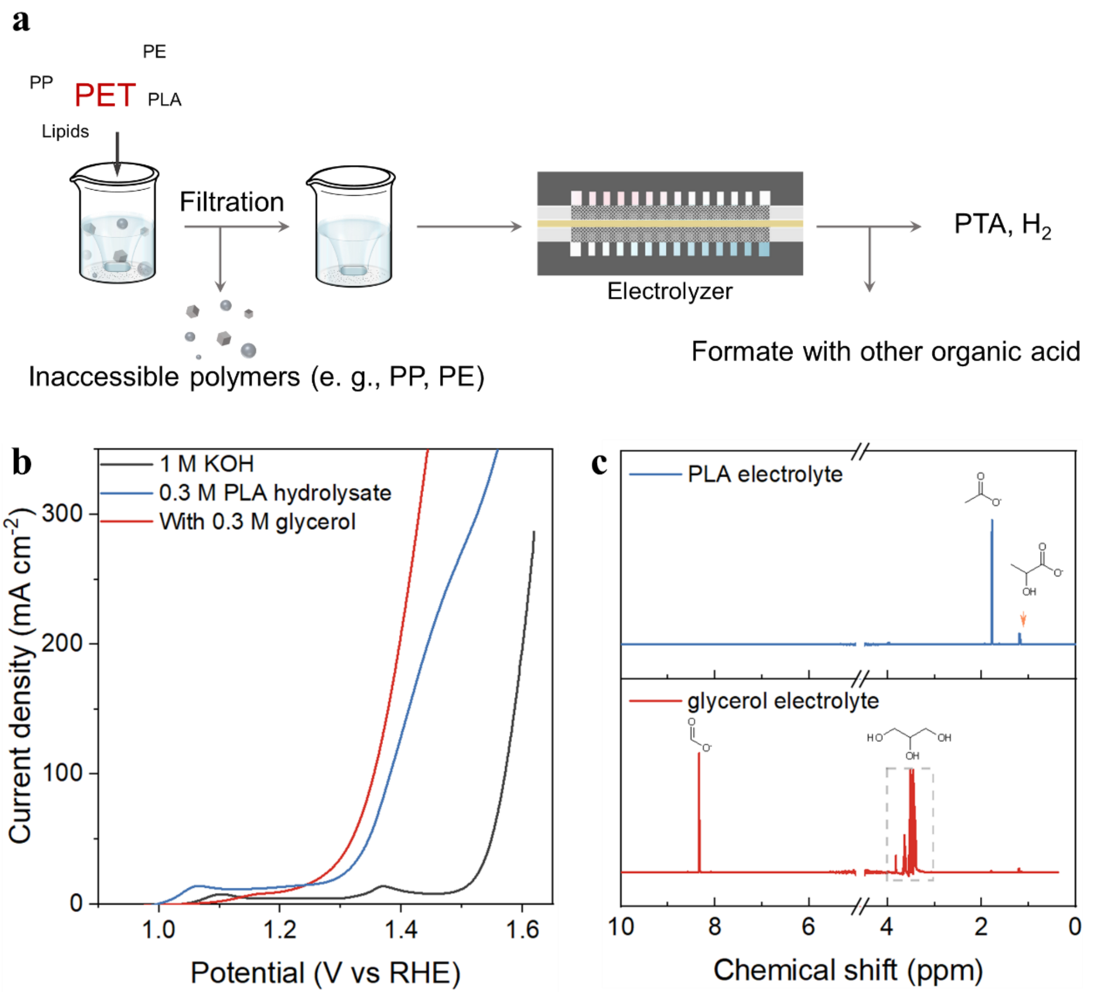

Supplementary Figure 27 a Schematic illustration of electrocatalytic upcycling of impure PET. **b** LSV curves (85% iR corrected) for electrooxidation of PLA hydrolysate and lipids-derived glycerol. **c** ^{1}H NMR spectra of PLA electrolyte and glycerol electrolyte.

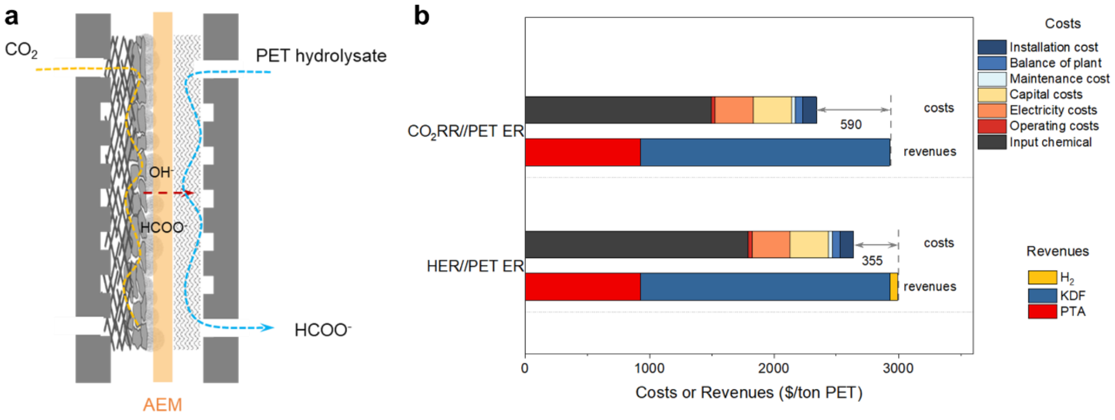

Supplementary Figure 28 a Concept of pairing CO_2RR and PET electroreforming (ER)**. b** TEA of HER//PET ER and CO_2RR//PET ER at 300 mA cm^{-2}.

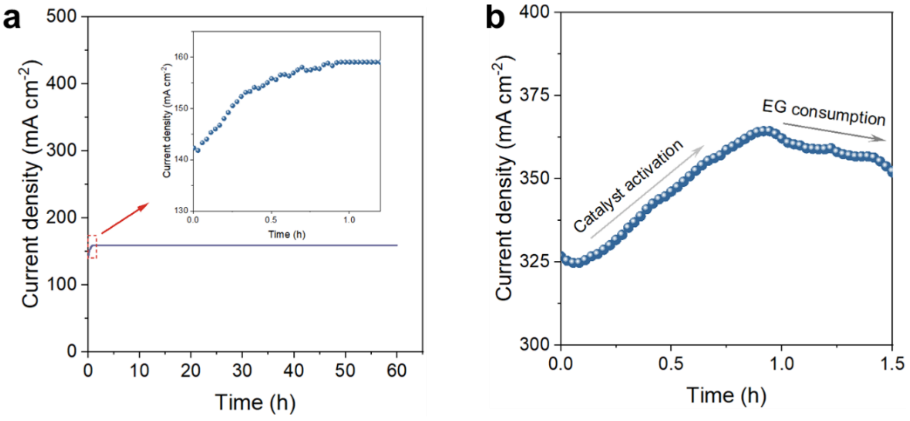

Supplementary Figure 29 a i-t curve for water splitting at 1.7 V vs RHE., the inset amplified figure suggests the activation of virgin $CoNi_{0.25}P$/NF. **b** i-t curve for EG oxidation over a virgin $CoNi_{0.25}P$/NF catalyst at 1.7 V vs RHE. The increase of current density in the initial 1 h is possibly ascribed to catalyst activation, while the decrease of current is induced by the consumption of EG in the electrolyte after 1 h. Reaction conditions: Pt foil (-)// $CoNi_{0.25}P$/NF(+), 1.7 V vs RHE.

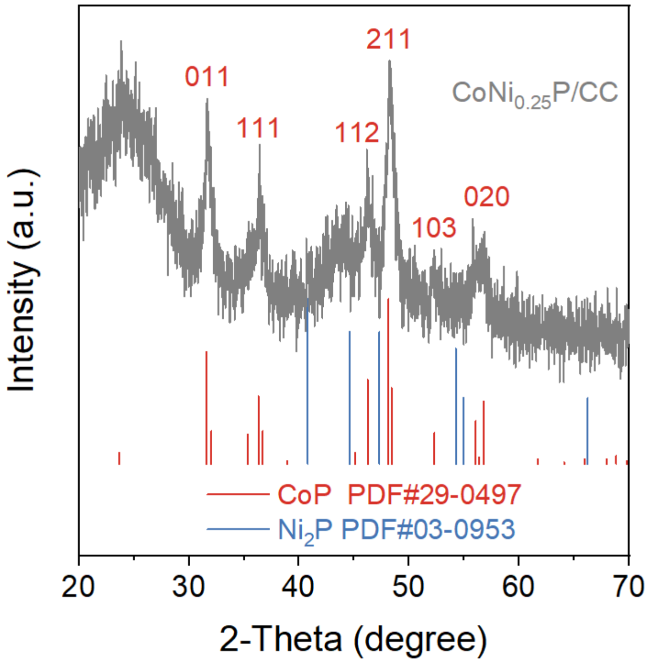

Supplementary Figure 30 XRD pattern of $CoNi_{0.25}P$/CC. This material was used for investigating the structural evolution of $CoNi_{0.25}P$ catalyst in HER, OER, and EG oxidation to eliminate the interference of nickel foam in XAFS, XRD, and XPS analysis.

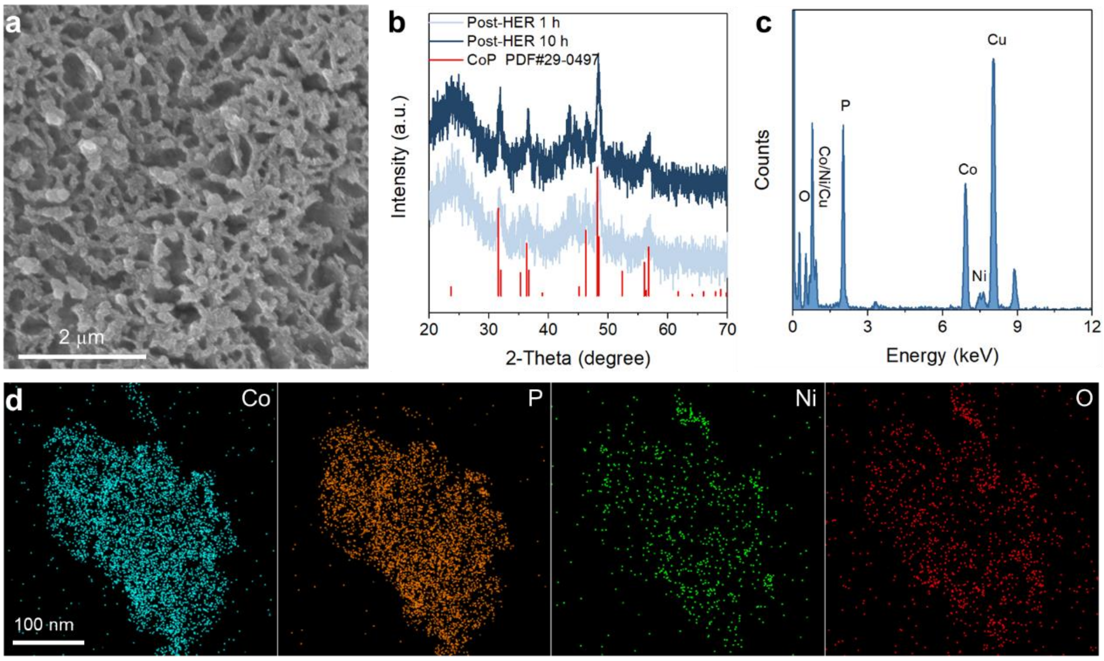

Supplementary Figure 31 Characterization of $CoNi_{0.25}P$ catalyst post-HER. **a** SEM image, **b** XRD pattern, **c** EDS spectrum, **d** elemental mapping of $CoNi_{0.25}P$ catalyst post-HER.

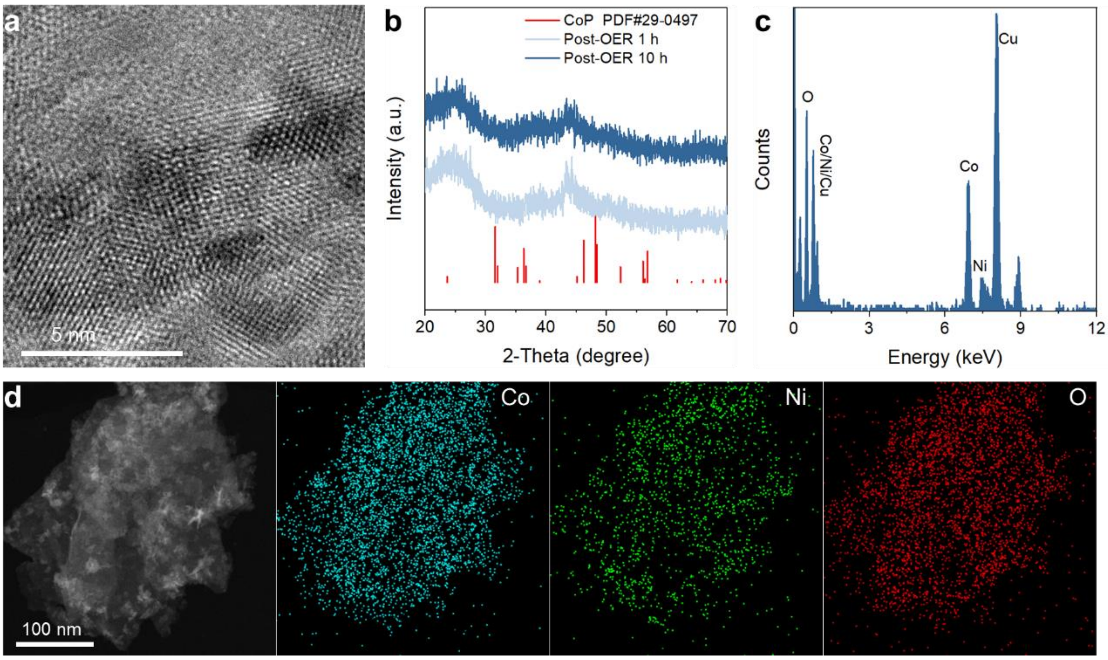

Supplementary Figure 32 Characterization of $CoNi_{0.25}P$ catalyst post-OER. **a** HR-TEM image, **b** XRD pattern, **c** EDS spectrum, **d** elemental mapping of $CoNi_{0.25}P$ catalyst post-OER.

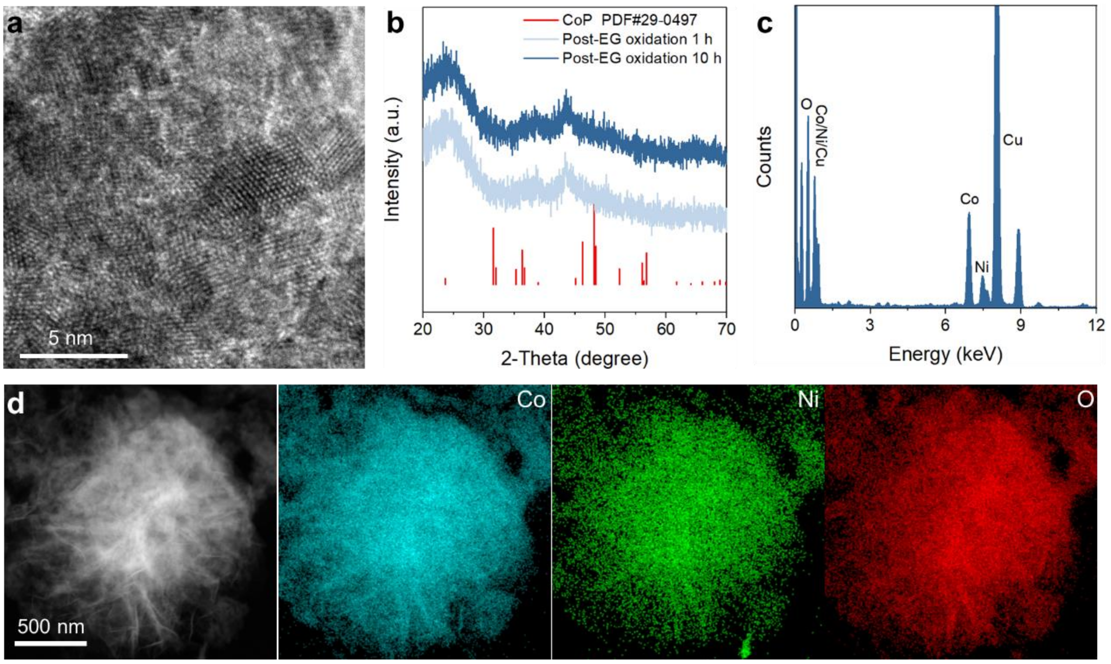

Supplementary Figure 33 Characterization of $CoNi_{0.25}P$ catalyst post-EG oxidation. **a** HR-TEM image, **b** XRD pattern, **c** EDS spectrum, **d** elemental mapping of $CoNi_{0.25}P$ catalyst post-EG oxidation.

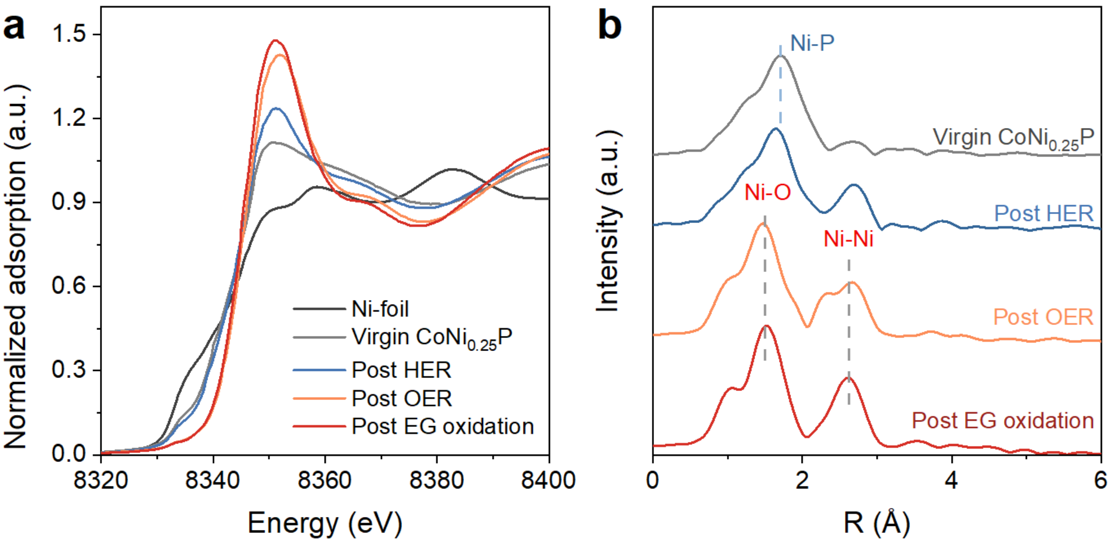

Supplementary Figure 34 XAFS profiles of Ni K-edge in $CoNi_{0.25}P$ materials. a Normalized Ni K-edge XANES spectra. **b** Fourier-transformed (FT) k^3-weighted Ni K-edge EXAFS spectra.

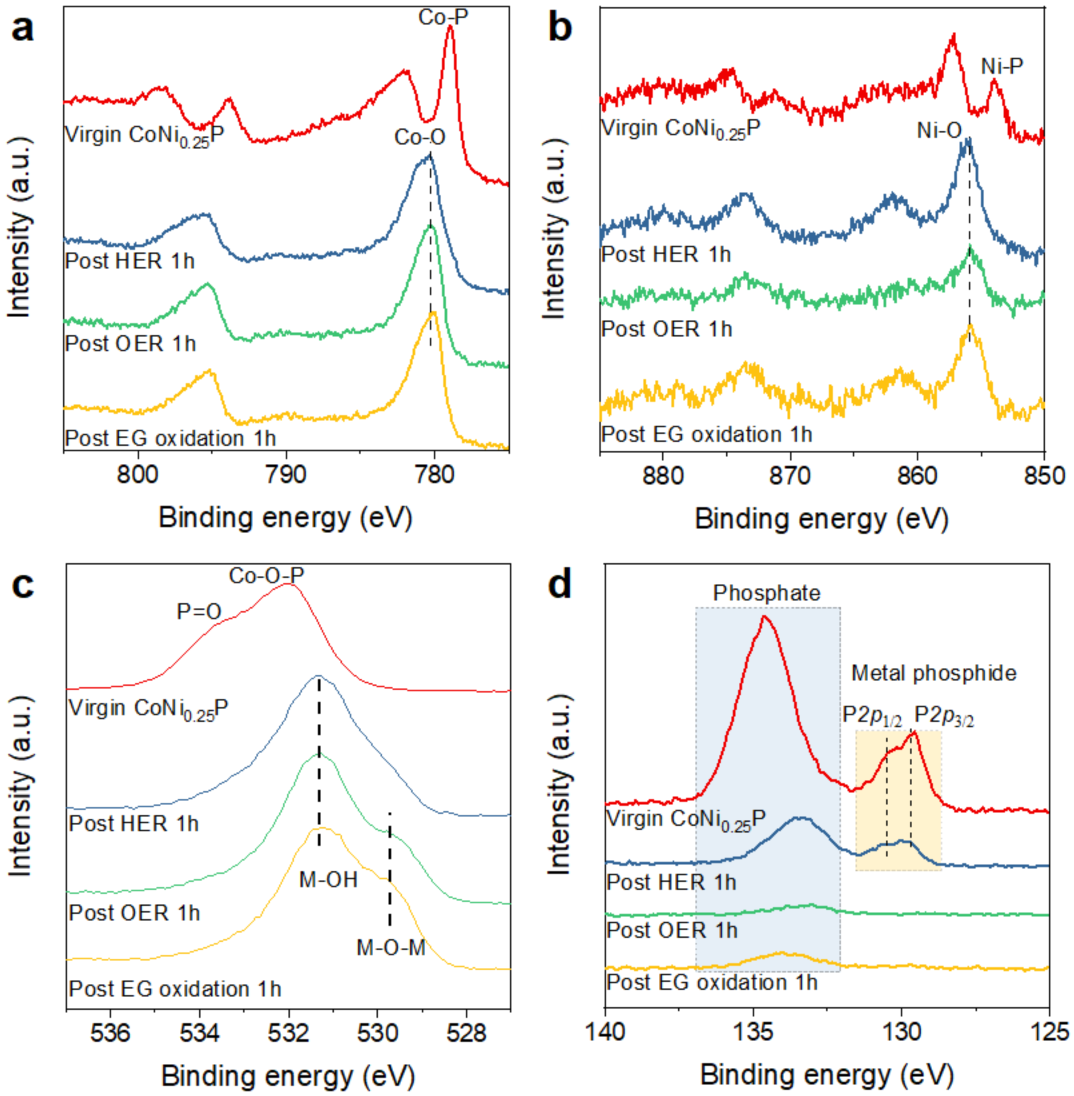

Supplementary Figure 35 XPS spectra of $CoNi_{0.25}P$ materials. **a** Co*2p* spectra. **b** Ni*2p* spectra. **c** O*1s* spectra. **d** P*2p* spectra.

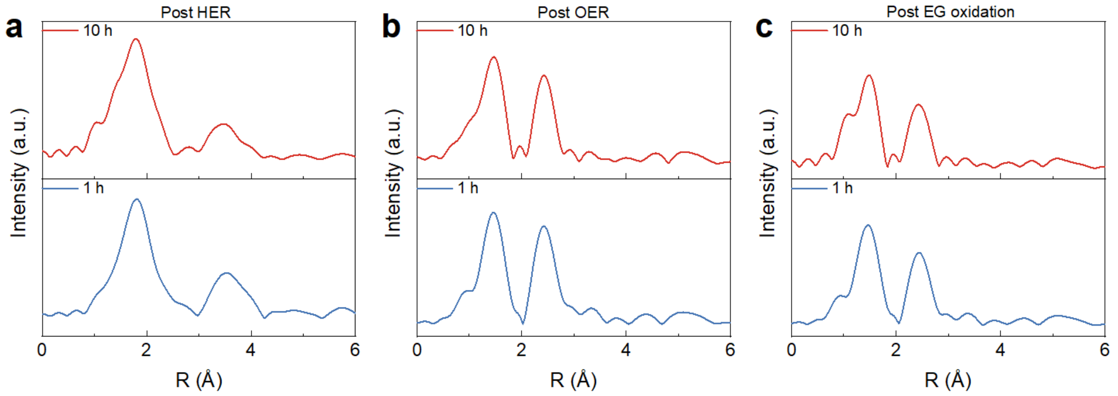

Supplementary Figure 36 Fourier-transformed (FT) k^3-weighted Co K-edge EXAFS spectra of $CoNi_{0.25}P$ catalyst after **a** HER, **b** OER, and **c** EG oxidation conditioned for 1 h and 10 h.

Supplementary Tables

Supplementary Table 1 Literatures of PET reforming toward H_2 and formate.

Catalyst	Energy input	Reaction conditions	$FE_{formate}$ (%)	Formate productivity (mmol cm^{-2} h^{-1})	H_2 productivity	Total TON of catalyst of long-term operation (mmol g^{-1}) [a]		Ref.
						Formate	H_2	
$CoNi_{0.25}P$	electricity	1 cm^{-2} electrode (2.85 mg/cm^{-2} metal phosphide), 1 M KOH with 0.3 M substrate	91.3	4.01	2215.4 mmol g^{-1} h^{-1}/ 6.31 mmol cm^{-2} h^{-1}	7.3×10^4	1.2×10^5	This work
CdS/CdO_x	solar	1 nmol catalyst, 10 M NaOH with 25 g/L substrate	Non-selective	-	12.4 mmol g^{-1} h^{-1}	-	~298	[1]
CN_x|Ni_2P	solar	40 mg catalyst, 1 M KOH with 25 g/L substrate	Non-selective	-	0.05 mmol g^{-1} h^{-1}	-	~1	[2]
Nano Ni-P	solar	1 cm^{-2} photoelectrode, 2 M KOH with 40 g/L substrate	~100	0.24	0.0031 mmol cm^{-2} h^{-1}	-	-	[3]

[a] TON: turnover number

Supplementary Table 2 Comparison of HER performance of $CoNi_{0.25}P$/NF with other non-noble metal catalysts in 1 M KOH.

**Catalyst**	**η_{10}** (mV)	**Tafel slope** (mV dec^{-1})	**Ref.**
$CoNi_{0.25}P$/NF	89.9	58.1	This work
Co_2P/CoNPC	208	83.9	[4]
B-CoP/CNT	56	69	[5]
Ni_5P_4-Ru	123	56.7	[6]
MoP@NCHSs	92	62	[7]
P-MoP/Mo_2N	54	47	[8]
MoO_2-FeP@C	103	48	[9]
CoP NFs	136	56.2	[10]
CoP@a-CoO_x	132	89	[11]
Ni foam/P-$CoMoO_4$	94	93	[12]
$CoNiPS_3$/C	136	60	[13]
CoP@ BCN	215	52	[14]
CoP/Co_2P	103	61.2	[15]
Ni_2P-Cu_3P	78	173	[16]
S-MoP NPL	104	56	[17]
$Co_{0.9}Ni_{0.1}Se$	185.7	58	[18]
$NiS_{0.5}Se_{0.5}$	70	78	[19]
m-$CoSe_2$	124	37	[20]
Co/β-Mo_2C@N-CNT	170	92	[21]

Supplementary Table 3 Structural parameters extracted from the XRD Rietveld refinement and EXAFS fitting of $CoNi_{0.25}P$ material.

XRD Rietveld refinement						
Phase	Composition (wt. %)	Atomic ratio of Co:Ni	a (Å)	b (Å)	c (Å)	Average M-P length (Å)

CoP	90.2	1:0.13	5.08	3.28	5.61	2.31±0.06
Ni_2P	9.8		5.74	5.74	3.50	2.23±0.04

EXAFS fitting

Path	R (Å)
Co-P	2.31±0.11
Ni-P	2.25±0.09

Supplementary Table 4 The concentration of metal ions in precursor solution and the final ratio of Co:Ni in the $CoNi_xP$ and Ni_2P materials.

Nominal designation [a]	Concentration of metal ions in precursor solution (mM) [b]		Determined atomic ratio of Co:Ni in the catalyst [c]	
	Co	Ni	ICP	EDX
CoP	100	-	1:0	1:0
$CoNi_{0.1}P$	100	10	1:0.07	1:0.09
$CoNi_{0.25}P$	100	25	1: 0.14	1: 0.17
$CoNi_{0.5}P$	100	50	1: 0.26	1: 0.23
Ni_2P	-	100	0	0

[a]**:** The designation of the materials is according to the ratio of Co/Ni in the metal precursor for electrodeposition.
[b]**:** The concentration of initial metal ions in electrolyte for electrodeposition.
[c]**:** The measured atomic ratio of Co:Ni in the obtained catalysts by ICP and EDX. Specifically, the $CoNi_xP$ nano-array on nickel foam was collected by sonification and further digested by concentrated HNO_3 for ICP analysis.

Supplementary Table 5 Comparison of catalytic performance of formic acid or formate production from waste.

**Substrate**	**Catalyst**	**Potential** (V vs RHE)	**Cell voltage** (V)	**Current density** (mA cm^{-1})	**FE** (%)	**Ref.**
EG	$CoNi_{0.25}P$/NF	-	1.77	500	81.4	This work
		1.7	-	350	90	

Methanol	Ni_3C	1.55	-	120	-	[22]
Methanol	$Co(OH)_2$@HOS/CP	-	1.50	10	100	[23]
		-	1.6	70	-	
Methanol	CuONS/CF	1.47	-	100	88.4	[24]
		-	1.52	50	91.3	
Methanol	$Ni(OH)_2$/NF	1.36	-	100	~100	[25]
		-	1.72	150	-	
Glycerol	$CuCo_2O_4$	1.3	-	10	89.1	[26]
Glycerol	Ni-Mo-N/CFC	-	1.36	10	95	[27]

Supplementary Table 6. Price of feedstocks and products.

Product	Price ($/ton)	Source
Feedstocks		
Waste PET	390	a
KOH	1280	[28]
H_2SO_4	45	[28]
H_2O	0.22	[28]
Formic acid	400	[29]
Products		
PTA	1260	[30]
EG	1400	[29]
KDF	1590	b
K_2SO_4	473	[28]
H_2	1900	[31]

a: The price of PET waste was assumed to be 40% of virgin PET. (https://jiage.molbase.cn/hangqing/PET)

b: Taken from online trade market. (https://jskolod.en.made-in-china.com/product/aFlJndtVYuch/China-Potassium-Diformate-98-.html)

Supplementary Note 1

To investigate the economic feasibility of this process for PET upcycling, we carried out a simplified techno-economic analysis using a model adapted from that of literatures reported by Sargent group.[31-33] The processing capacity of the plant is 200 ton of waste PET per day. Supplementary Figs. 1-2 summarize the model used to calculate the plant-gate levelized cost of processing PET ($/ton PET) at different current density (50, 100, and 300 mA cm^{-2}). The price of input chemicals and products were listed in Supplementary Table 6.

Below is the list of assumptions made for the calculations.

1. The capital costs of electrolyzer is sensitive to the operating current density, we assume a cost of $10,000 per m^2 of electrolyzer. The total catalyst and membrane cost are 5 % of the electrolyzer cost.[31]

2. The capital costs of hydrolyzer and distillation equipment are dependent on the process capacity of PET. Their combined cost is assumed to be 50% of PET feedstock cost.

3. The capacity factor is expected to be operational on any given day, is assumed to be 0.8, which means the plant will be operational 19.2 hours a day.[31]

4. Input chemicals include PET, potassium hydroxide, formic acid and water. The output products include KDF, PTA, and H_2. The waste PET contains ~15% of unreacted impurities.[30]

5. The faradaic efficiency and selectivity to formate from EG is 80%, and the faradaic efficiency to H_2 is assumed to be 100 %.[32] The KDF yield from formate is assumed to be 70%.

6. The price of electricity is assumed to be 10 ¢/kWh.[31] The electricity costs comprise 3 components, electrolyzer for EG and water electrolysis, hydrolyzer for PET hydrolysis, separation equipment for distillation and drying. The electricity costs for hydrolysis and products separation are assumed to be equal to electrolysis.

7. Both operation and maintenance costs are assumed to be 10% of the capital costs.

The calculation process

1. Capital costs

The electrolyser cost. According to the current required and the assumed operating current density of 300 mA cm^{-2}, we can calculate the area of electrolyser needed:

$$\text{Area of electrolyser} = \frac{\text{I}}{\text{i}}(\text{m}^2)$$

Where i is current density.

The electrolyser cost can be calculated based on the estimate of $10000 per m^2, therefore:

$$\text{Cost of electrolyser} = \text{Area of electrolyser} \times \$10000 \text{ per m}^2$$

The total catalyst and membrane cost are assumed to be 5 % of electrolyzer cost and is calculated as:

$$\text{Cost of catalyst and membrane} = \text{Cost of electrolyser} \times 5\%$$

The capital costs of hydrolyzer and distillation equipment are assumed to be 50% of PET and are calculated as:

$$\text{The capital costs of hydrolyzer and distillation equipment} = \text{Cost of PET} \times 50\%$$

Therefore, the capital costs component can be calculated as:

$$\text{Capital costs} = \frac{\text{The electrolyser cost} + \text{The catalyst and membrane costs}}{\text{Lifetime of plant}} + \text{The hydrolyzer and distillation equipment costs}$$

2. Maintenance cost

This is assumed to be 10% of the capital costs and is calculated as:

$$\text{Maintenance cost per day} = \text{The capital cost} \times 10\%$$

3. Balance of plant

$$\text{Balance of plant} = \text{The capital cost} \times \text{Balance of plant factor}$$

4. Installation costs

$$\text{Installation cost} = \text{The capital cost} \times \text{Lang factor}$$

5. Electricity costs:

Total charge required to treatment per ton of PET can be calculated as follows:

$$Q = \frac{\text{Mass of ethylene glycol（g）} \times F \times N}{\text{Molar mass of ethylene glycol}\left(\frac{g}{mol}\right) \times \text{Faradaic efficiency}}$$

Where Q is the total charge, F is the Faraday's constant and N takes the value 2 since ethylene glycol oxidation to formic acid is a six-electron transfer process.

The current required to sustain this process can be calculated as follows, with a capacity factor of 0.8:

$$I = \frac{Q}{\text{Time in a day(s)} \times \text{Capacity factor}}$$

Where I is the current.

The power required to sustain this process can be calculated as follows, assuming an operating cell potential of 1.7 V:

$$P = \frac{U \times I}{1000} (kW)$$

The energy use per day can be calculated as follows:

$$\text{Energy use per day(kWh)} = P \times \text{Time in a day(h)} \times \text{Capacity factor}$$

The electrolyzer electricity cost of per day can be calculated as follows:

$$\text{Electrolyser electricity cost of per day} = \text{Energy use per day} \times \text{Cost per kWh.}$$

The electricity costs for hydrolysis and products separation are assumed to be equal to electrolysis and are calculated as:

$$\text{Hydrolysis and products separation electricity costs of per day} = \text{Electrolyser electricity cost of per day}$$

Therefore, the electricity cost per day can be calculated as:

$$\text{Electricity costs per day} = \text{Electrolyser electricity cost of per day} + \text{Hydrolysis and products separation electricity costs of per day}$$

6. Input chemicals costs：Materials require 1 ton of PET, 828 kg KOH, 1032.5 kg formic acid and 333.3 kg water.

$$\text{Input chemicals} = \text{Cost of PET} \times \text{Mass of PET needed} + \text{Cost of KOH} \times \text{Mass of KOH needed} + \text{Cost of formic acid} \times \text{Mass of formic acid needed} + \text{Cost of water} \times \text{Mass of water needed}。$$

7. Operating costs

This is assumed to be 10% of the capital costs and is calculated as:

$$\text{Operating cost per day} = \text{The capital cost} \times 10\%$$

Total plant gate levelized cost

Finally, the total cost can be calculated by adding up all 7 components:

$$\text{Total costs} = \text{Input chemicals} + \text{Electricity cost} + \text{Capital cost} + \text{Operating cost} + \text{Maintenance cost} + \text{Balance of plant cost} + \text{Installation cost}$$

The products of this process include terephthalic acid (PTA), potassium dicarboxylate (KDF). Per ton of PET as raw material can obtain 735.5 kg PTA and 1344.3 kg KDF. Therefore, the product value can be calculated as:

$$\text{Product value} = \text{Cost of PTA} \times \text{ Mass of PET obtained} + \text{Cost of KDF} \times \text{Mass of KOH obtained}。$$

The profit per day from this process can be calculated as:

$$\text{Profit per ton of PET} = \text{Product value} - \text{Total costs}$$

Cathode hydrogen evolution, which has a 100% faradaic efficiency.

$$\text{Mass of hydrogen produced per day} = \frac{\text{Q} \times \text{Molar mass of hydrogen}}{\text{N} \times \text{F}}$$

Profit from hydrogen can be calculated as:

$$\text{Profit from hydrogen per ton of PET} = \text{Cost of } H_2 \times \text{Mass of } H_2 \text{ obtained}$$

Therefore, total profit per ton PET can be calculated as:

$$\text{Total profit per tonne of PET} = \text{Profit per ton of PET} + \text{Profit from hydrogen per ton of PET}$$

Supplementary Note 2

To investigate the economic feasibility of pairing electrocatalytic carbon dioxide reduction reaction (CO_2RR) to formate and PET upcycling, TEA was carried out based on the following assumptions.

1. The operating current density is 300 mA cm^{-2}.
2. The faradaic efficiency to formate from CO_2 is 80%.
3. The cost for producing formic acid from CO_2 is 185 $/ton.[29]

Supplementary References

1. Uekert T, Kuehnel MF, Wakerley DW, Reisner E. Plastic waste as a feedstock for solar-driven H_2 generation. *Energy Environ. Sci.* **11**, 2853-2857 (2018).
2. Uekert T, Kasap H, Reisner E. Photoreforming of Nonrecyclable Plastic Waste over a Carbon Nitride/Nickel Phosphide Catalyst. *J. Am. Chem. Soc.* **141**, 15201-15210 (2019).
3. Lin C-Y, Huang S-C, Lin Y-G, Hsu L-C, Yi C-T. Electrosynthesized Ni-P nanospheres with high activity and selectivity towards photoelectrochemical plastics reforming. *Appl. Catal. B-Environ.* **296**, 120351 (2021).
4. Liu H*, et al.* Metal-Organic-Framework-Derived Co2P Nanoparticle/Multi-Doped Porous Carbon as a Trifunctional Electrocatalyst. *Adv. Mater.* **32**, e2003649 (2020).
5. Cao E*, et al.* Boron-Induced Electronic-Structure Reformation of CoP Nanoparticles Drives Enhanced pH-Universal Hydrogen Evolution. *Angew. Chem. Int. Ed.* **59**, 4154-4160 (2020).
6. He Q*, et al.* Achieving Efficient Alkaline Hydrogen Evolution Reaction over a Ni5 P4 Catalyst Incorporating Single-Atomic Ru Sites. *Adv. Mater.* **32**, e1906972 (2020).
7. Zhao D*, et al.* Synergistically Interactive Pyridinic-N-MoP Sites: Identified Active Centers for Enhanced Hydrogen Evolution in Alkaline Solution. *Angew. Chem. Int. Ed.* **59**, 8982-8990 (2020).
8. Gu Y*, et al.* Two-Dimensional Porous Molybdenum Phosphide/Nitride Heterojunction Nanosheets for pH-Universal Hydrogen Evolution Reaction. *Angew. Chem. Int. Ed.* **60**, 6673-6681 (2021).
9. Yang G*, et al.* Interfacial Engineering of MoO_2-FeP Heterojunction for Highly Efficient Hydrogen Evolution Coupled with Biomass Electrooxidation. *Adv. Mater.* **32**, e2000455 (2020).
10. Ji L, Wang J, Teng X, Meyer TJ, Chen Z. CoP Nanoframes as Bifunctional Electrocatalysts for Efficient Overall Water Splitting. *ACS Catal.* **10**, 412-419 (2019).
11. Yu J*, et al.* Bifunctionality from Synergy: CoP Nanoparticles Embedded in Amorphous CoOx Nanoplates with Heterostructures for Highly Efficient Water Electrolysis. *Adv. Sci.* **5**, 1800514 (2018).
12. Zhao S*, et al.* The Role of Phosphate Group in Doped Cobalt Molybdate: Improved Electrocatalytic Hydrogen Evolution Performance. *Adv. Sci.* **7**, 1903674 (2020).
13. Liang QH*, et al.* Mosaic-Structured Cobalt Nickel Thiophosphate Nanosheets Incorporated N-doped Carbon for Efficient and Stable Electrocatalytic Water Splitting. *Adv. Funct. Mater.* **28**, (2018).
14. Tabassum H*, et al.* Metal-Organic Frameworks Derived Cobalt Phosphide Architecture Encapsulated into B/N Co-Doped Graphene Nanotubes for All pH Value Electrochemical Hydrogen Evolution. *Adv. Energy Mater.* **7**, 1601671 (2017).
15. Chen L, Zhang Y, Wang H, Wang Y, Li D, Duan C. Cobalt layered double hydroxides derived CoP/Co_2P hybrids for electrocatalytic overall water splitting. *Nanoscale* **10**, 21019-21024 (2018).
16. Yu L*, et al.* In Situ Growth of Ni_2P–Cu_3P Bimetallic Phosphide with Bicontinuous Structure on Self-Supported NiCuC Substrate as an Efficient Hydrogen Evolution Reaction Electrocatalyst. *ACS Catal.* **9**, 6919-6928 (2019).
17. Liang K*, et al.* S-Doped MoP Nanoporous Layer Toward High-Efficiency Hydrogen Evolution in pH-Universal Electrolyte. *ACS Catal.* **9**, 651-659 (2018).

18. Zhong W, *et al.* Coupled Vacancy Pairs in Ni-Doped CoSe for Improved Electrocatalytic Hydrogen Production Through Topochemical Deintercalation. *Angew. Chem. Int. Ed.* **59**, 22743-22748 (2020).
19. Wang Y, *et al.* Lattice-Strain Engineering of Homogeneous $NiS_{0.5}Se_{0.5}$ Core-Shell Nanostructure as a Highly Efficient and Robust Electrocatalyst for Overall Water Splitting. *Adv. Mater.* **32**, e2000231 (2020).
20. Zhang XL, *et al.* Polymorphic cobalt diselenide as extremely stable electrocatalyst in acidic media via a phase-mixing strategy. *Nat. Commun.* **10**, 5338 (2019).
21. Ouyang T, Ye YQ, Wu CY, Xiao K, Liu ZQ. Heterostructures Composed of N-Doped Carbon Nanotubes Encapsulating Cobalt and beta-Mo2 C Nanoparticles as Bifunctional Electrodes for Water Splitting. *Angew. Chem. Int. Ed.* **58**, 4923-4928 (2019).
22. Li J, *et al.* Selective Methanol-to-Formate Electrocatalytic Conversion on Branched Nickel Carbide. *Angew. Chem. Int. Ed.* **59**, 20826-20830 (2020).
23. Xiang K, *et al.* Boosting H_2 Generation Coupled with Selective Oxidation of Methanol into Value-Added Chemical over Cobalt Hydroxide@Hydroxysulfide Nanosheets Electrocatalysts. *Adv. Funct. Mater.* **30**, 1909610 (2020).
24. Wei X, Li Y, Chen L, Shi J. Formic acid electro-synthesis by concurrent cathodic CO_2 reduction and anodic CH_3OH oxidation. *Angew. Chem. Int. Ed.* **60**, 3148-3155 (2021).
25. Hao J, *et al.* In situ facile fabrication of $Ni(OH)_2$ nanosheet arrays for electrocatalytic co-production of formate and hydrogen from methanol in alkaline solution. *Appl. Catal. B-Environ.* **281**, 119510 (2021).
26. Han XT, *et al.* Electrocatalytic oxidation of glycerol to formic acid by $CuCo_2O_4$ spinel oxide nanostructure catalysts. *ACS Catal.* **10**, 6741-6752 (2020).
27. Li Y, Wei X, Chen L, Shi J, He M. Nickel-molybdenum nitride nanoplate electrocatalysts for concurrent electrolytic hydrogen and formate productions. *Nat. Commun.* **10**, 5335 (2019).
28. Liu WJ, *et al.* Efficient electrochemical production of glucaric acid and H_2 via glucose electrolysis. *Nat. Commun.* **11**, 265 (2020).
29. Bushuyev OS, *et al.* What Should We Make with CO2 and How Can We Make It? *Joule* **2**, 825-832 (2018).
30. Uekert T, Pichler CM, Schubert T, Reisner E. Solar-driven reforming of solid waste for a sustainable future. *Nat. Sustain.* **4**, 383-391 (2020).
31. Leow WR, *et al.* Chloride-mediated selective electrosynthesis of ethylene and propylene oxides at high current density. *Science* **368**, 1228-1233 (2020).
32. Lum Y, *et al.* Tuning OH binding energy enables selective electrochemical oxidation of ethylene to ethylene glycol. *Nat. Catal.* **3**, 14-22 (2020).
33. De Luna P, Hahn C, Higgins D, Jaffer SA, Jaramillo TF, Sargent EH. What would it take for renewably powered electrosynthesis to displace petrochemical processes? *Science* **364**, eaav3506 (2019).
